# Ampere-level CO_2_ electroreduction with single-pass conversion exceeding 85% in acid over silver penetration electrodes

**DOI:** 10.1038/s41467-024-50521-8

**Published:** 2024-07-19

**Authors:** Shoujie Li, Xiao Dong, Gangfeng Wu, Yanfang Song, Jianing Mao, Aohui Chen, Chang Zhu, Guihua Li, Yiheng Wei, Xiaohu Liu, Jiangjiang Wang, Wei Chen, Wei Wei

**Affiliations:** 1 Low-Carbon Conversion Science and Engineering Center, Shanghai Advanced Research Institute, Chinese Academy of Sciences, Shanghai, China; 2 State Key Laboratory of Low Carbon Catalysis and Carbon Dioxide Utilization, Shanghai Advanced Research Institute, Chinese Academy of Sciences, Shanghai, China; 3 Shanghai Institute of Applied Physics, Chinese Academy of Sciences, Shanghai, China; 4 School of Physical Science and Technology, ShanghaiTech University, Shanghai, China

**Keywords:** Electrocatalysis, Electrocatalysis, Sustainability, Electrocatalysis

## Abstract

Synthesis of valuable chemicals from CO_2_ electroreduction in acidic media is highly desirable to overcome carbonation. However, suppressing the hydrogen evolution reaction in such proton-rich environments remains a considerable challenge. The current study demonstrates the use of a hollow fiber silver penetration electrode with hierarchical micro/nanostructures to enable CO_2_ reduction to CO in strong acids via balanced coordination of CO_2_ and K^+^/H^+^ supplies. Correspondingly, a CO faradaic efficiency of 95% is achieved at a partial current density as high as 4.3 A/cm^2^ in a pH = 1 solution of H_2_SO_4_ and KCl, sustaining 200 h of continuous electrolysis at a current density of 2 A/cm^2^ with over 85% single-pass conversion of CO_2_. The experimental results and density functional theory calculations suggest that the controllable CO_2_ feeding induced by the hollow fiber penetration configuration primarily coordinate the CO_2_/H^+^ balance on Ag active sites in strong acids, favoring CO_2_ activation and key intermediate *COOH formation, resulting in enhanced CO formation.

## Abstract (web summary)

Suppression of the competing H_2_ evolution reaction in CO_2_ electroreduction in strong acids remains a considerable challenge. Here, authors report a silver-based electrode that enables CO_2_ conversion to CO exceeding 85% at ampere-level current densities in pH = 1 electrolytes.

## Introduction

The electrochemical conversion of CO_2_ driven by renewable electricity can produce value-added chemicals and feedstocks while mitigating CO_2_ emissions^1–3^. Numerous efforts have been made to develop catalysts with high current density (*j* > 1 A/cm^2^) and high faradaic efficiency (FE > 90%) toward CO, formate, etc^4–9^. Typically, alkaline or neutral electrolytes are used to suppress the competing hydrogen evolution reaction (HER) while promoting the electrocatalytic CO_2_ reduction reaction (CO_2_RR)^10–12^. However, during CO_2_RR and HER, the rapid consumption of H^+^ creates a locally alkaline environment close to the catalyst surface. Consequently, rather than being reduced, a major fraction of the input CO_2_ is consumed in the electrolyte via reaction with hydroxide ion (OH^−^) to produce (bi)carbonate^13–17^. In addition, transporting (bi)carbonate to the cathode flow field or anode results in a significant reduction of locally available CO_2_ and a low CO_2_ single-pass carbon efficiency (SPCE), impeding the practical applications of CO_2_ electrolysis^18–21^.

One strategy for addressing these issues is conducting CO_2_RR in an acidic medium^22–28^. That is, in a catholyte with a low pH, when the hydronium (H_3_O^+^) serves as the proton source for CO_2_RR and HER, no hydroxide ion (OH^–^) will be generated, and CO_2_ conversion can proceed without (bi)carbonate formation; even when H_2_O is the proton source, any OH^–^ or (bi)carbonate generated locally will be neutralized or converted back to CO_2_ by protons in the bulk electrolyte, preventing CO_2_ from transferring to the anode^22,29^. However, efficient CO_2_RR in an acidic medium is difficult due to the kinetically superior HER outcompeting the reduction of CO_2_^23,30,31^. For instance, in a strong acid with a pH ≤ 1, the FE of the CO_2_RR product is nearly close to zero^23^. One of the main reasons is that the adsorbed hydrogen (*H) acts as an intermediate for HER, out-competing the adsorption of CO_2_ (*CO_2_) overactive sites during CO_2_RR in an acidic medium^23,30,31^. Recently, it was discovered that K^+^ in the electrolyte could shield the electrode electric field and inhibit the transport of hydrogen ions (H^+^) and that the rapid consumption of surface H^+^ at high *j* could increase the pH near active sites, allowing for efficient CO_2_RR^22–25^. Several electrocatalysts, including Au^24^, Ag^32^, Cu^24^, and Ni_5_@NCN^33^, have demonstrated the ability to CO_2_RR in acid, but their high CO_2_RR selectivity (FE_CO2RR_ > 80%) could only be achieved in a limited range of *j* (≤0.5 A/cm^2^) with a low rate of product formation, which hinders their scalable applications.

**Fig. 1:**
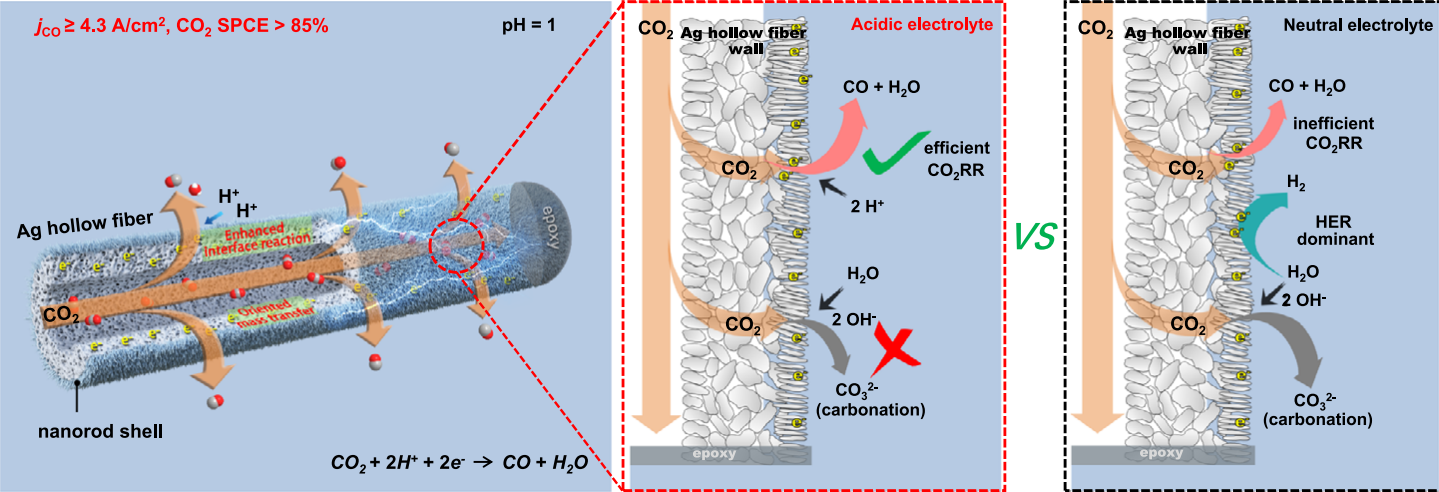
Function outline of acidic CO_2_ electroreduction over hollow fiber silver electrode. Schematic of Ag hollow fiber penetration electrode for boosting CO_2_ electroreduction to CO in a strongly acidic electrolyte (pH = 1) and the left schematic illustration of Ag hollow fiber was reproduced with permission from the reference^37^. Copyright [2022] [Springer Nature].

Recently, a hollow fiber penetration electrode (HPE) with a compact structure has shown promising potential for high-rate and efficient CO_2_ reduction due to enhanced mass transport^34–39^. The unique three-dimensional electrode structure compels gaseous CO_2_ to permeate its abundant pores; the adequate oriented mass transfer at extensive triphasic reaction interfaces significantly improves the electrocatalytic kinetics. Herein, an Ag_2_CO_3_-derived hierarchical micro/nanostructured silver HPE (CD-Ag HPE) was used to investigate the effects of catalyst microenvironments (such as local concentrations of K^+^/H^+^ and CO_2_) on CO_2_ electrolysis performance in an acidic medium (pH = 1) (Fig. 1). By optimizing catholyte composition (H^+^, K^+^ concentration) and input CO_2_ flow rate, a high CO current density (*j*_CO_) of 4.3 A/cm^2^ with CO FE of 95% and stable electrolysis of 200 h at 2 A/cm^2^ were achieved in a strongly acidic electrolyte. Furthermore, a CO_2_ SPCE of over 85% was achieved at 2 A/cm^2^ by modulating the availability of CO_2_. In addition, the density functional theory (DFT) calculations indicated that the coexistence of H^+^ and K^+^ localized around the Ag sites played a crucial role in the formation of the key intermediates *COOH and *H, which not only suppressed the competitive HER but also promoted the CO_2_RR.

## Results and discussion

### Electrode preparation and characterization

**Fig. 2:**
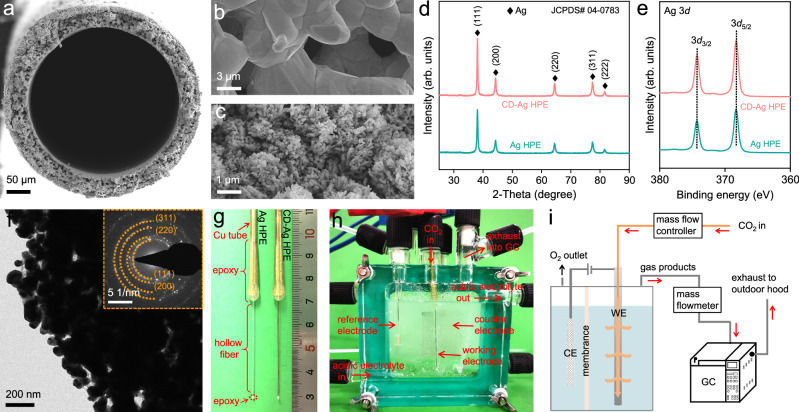
Structural and compositional characterization. SEM images of (**a**) cross section and (**b**), (**c**) outer surface of (**a**), (**c**) CD-Ag HPE and **b** Ag HPE. **d** XRD patterns, and **e** XPS spectra of Ag HPE and CD-Ag HPE. **f** TEM image and corresponding SAED pattern (insert of **f**) of CD-Ag HF. Optical images of the (**g**) working electrode of Ag HPE and CD-Ag HPE, (**h**) electrolyte flow two-compartment electrolysis cell from a side view. **i** Schematic illustration of the electrolysis system for CO_2_ electroreduction. WE: working electrode, CE: counter electrode, GC: gas chromatograph.

The CD-Ag HPE was fabricated via a two-step approach^37^ that was based on commercial Ag powder; additionally, it included an industrially viable phase-inversion/sintering process to obtain Ag HPE and an electrochemical redox process to obtain CD-Ag HPE (Supplementary Fig. 1). Compared to Ag HPE, CD-Ag HPE was about 20 μm thick, with partially ordered nanorods evenly coating the outer surface (Fig. 2a–c and Supplementary Fig. 2). This unique hierarchical micro/nanostructured architecture provided an increased electrochemical active surface area (ECSA, Supplementary Fig. 3) that maximized the three-phase reaction interfaces and enabled the efficient transport of reactants and products to/from the active sites for high-efficiency electrocatalytic reaction^34–39^. The X-ray diffraction (XRD) patterns were indexed to metallic Ag (111), (200), (220), (311), and (222) planes (JCPDS no. 04-0783), and there was no obvious crystal face orientation, which was almost the same as those of commercial Ag powder (Fig. 2d and Supplementary Figs. 4a, b). The X-ray photoelectron spectroscopy (XPS) confirmed that the surface compositions of CD-Ag HPE were identical to that of metallic silver (Fig. 2e and Supplementary Fig. 4c). In addition, the selected-area diffraction (SAED) pattern (inset of Fig. 2f) of CD-Ag HPE agreed well with the XRD results. Supplementary Fig. 5 depicted the high-resolution transmission electron microscopy (HRTEM) image and the corresponding fast Fourier transform (FFT) pattern of the marked region, in which only metallic Ag was observed.

### Electrocatalytic CO_2_RR in neutral electrolyte

The electrochemical experiment of the CO_2_RR was conducted in two chamber electrolysis cell with a three-electrode system at room temperature, where the CD-Ag HPE was used as the working electrode and gas diffuser (Fig. 2g–i). During CO_2_ electroreduction, CO_2_ penetrated through the porous wall of the CD-Ag HPE into the electrolytes via the copper tube, forming a large amount of bubbles. This unique oriented mass transfer of CO_2_ could induce the in-situ formation of extensive dynamic CO_2_(gas)–liquid–catalyst triphasic reaction interfaces, which significantly improve the mass transfer of CO_2_, electrons, protons, products as well as CO_2_RR kinetics^34–39^. Subsequently, a mixture of CO produced by CO_2_RR, H_2_ produced by HER, and unreacted CO_2_ flows out through an outlet connected to the top right of the electrolysis cell. The actual outlet flow rate was measured by an independent mass flowmeter and then sent to online gas chromatography (GC) for quantification. And the exhaust from the GC was vented to the outdoor hood (Fig. 2h, i). Unless otherwise specified, a flow rate of 30 standard cubic centimeters per minute (sccm) was used to compare our performance to that of other studies^34–36^. It was shown that only CO and H_2_ were detected at the *j* range of 0.1–4.0 A/cm^2^ in a neutral catholyte (pH ≈ 6.6) or in a strong acidic catholyte (Supplementary Figs. 6–9, pH = 1). The H_2_ FE remained below 5% as *j* increased up to 2.0 A/cm^2^, whereas the CO FE remained as high as 90.0% at *j* of 2.5 A/cm^2^, resulting in a high *j*_CO_ of 2.3 A/cm^2^. Such CO_2_RR to CO performance in neutral catholyte distinguishes these electrocatalysts from other recent prominent electrocatalysts (Supplementary Fig. 6 and Supplementary Table 1). Subsequently, the CO FE decreased rapidly with increasing *j*, falling to 72.2% at 4.0 A/cm^2^, indicating an increase in HER at further elevated *j* values. Notably, During both CO_2_RR and HER, the consumption of H^+^ will likely produce a locally alkaline environment close to the catalyst surface. As a result, we hypothesized that at high *j* in a neutral electrolyte, rather than being reduced, a portion of the input CO_2_ would instead be consumed in the electrolyte through a reaction with OH^−^ to produce (bi)carbonate. Consequently, local CO_2_ levels are inadequate and HER growth is kinetically more favorable.

### Effects of H^+^ concentration and CO_2_ carbonation on CO_2_RR

**Fig. 3:**
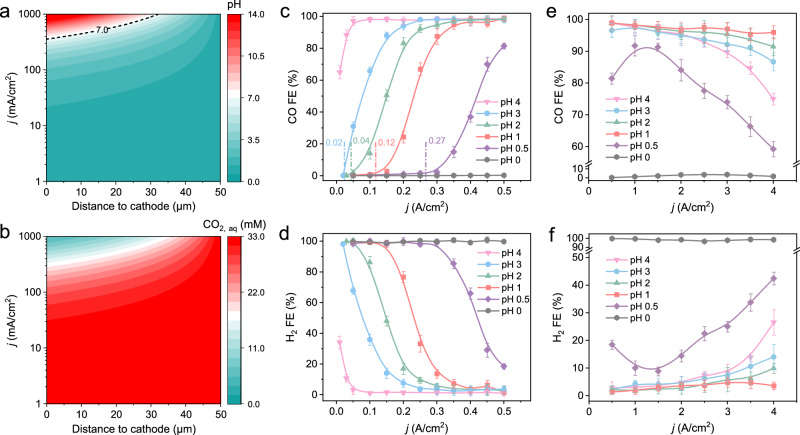
Effects of H^+^ concentration and CO_2_ carbonation on CO_2_RR. Modeling of (**a**) pH, (**b**) concentration profile of CO_2_ at different distances to cathode and *j* in 0.05 M H_2_SO_4_ and 3 M KCl. **c**, **e** CO and **d**, **f** H_2_ FE over CD-Ag HPE as a function of applied *j* measured in 3 M KCl and H_2_SO_4_ catholytes with different pH values. The input CO_2_ flow rate was 30 sccm. The error bars in (**c**)–(**f**) represent one standard deviation based on five independent tests.

The calculations based on the reaction and diffusion of species model (Supplementary Fig. 10) within a typical diffusion layer of 50 μm showed that, in the presence of K^+^ in a strong acidic electrolyte (pH = 1), the surface pH (distance to cathode of 0 μm) was similar to the bulk at *j* < 100 mA/cm^2^, while became neutral or basic when current density increase further due to the consumption rate of local protons that exceeds mass transport of protons from the bulk (Fig. 3a, b, *j* > 100 mA/cm^2^), effective CO_2_RR would dominate while HER was inhibited (Figs. 3c, d). Thus, the proton source of CO_2_RR comes from water on the electrode surface, despite the bulk pH still being in an acidic range (Fig. 3a). As shown in Fig. 3a, although the pH at the cathode surface rapidly increased with the *j* increase, the pH still remained in an acidic range with the increase in the distance from the cathode. When at a high *j* of 1 A/cm², although the surface is alkaline, the pH decreased to 7 at 30 μm away from the cathode. In comparison, similar conditions (pH 7 and pH 10.5 at a distance to the cathode of 40 μm for bulk pH 4 and 7, respectively) were reached at much lower *j* (about 200 mA/cm^2^) in electrolytes of pH 4 and 7 (Supplementary Fig. 11b, c). That means at high *j*, the carbonate formation would be more serious in an electrolyte with insufficient H^+^. In addition, the corresponding variation of CO_2_ concentration distribution showed that, even in a strong acidic electrolyte with pH 1 (Fig. 3b), the surface available CO_2_ concentration decreased gradually with the *j* increased above 200 mA/cm^2^, which is mainly due to the dual effects of the conversion of CO_2_ and the carbonation of CO_2_ caused by the increasing of pH^22,23^. However, the available CO_2_ concentration increased to a high level within about 30 μm of the cathode, even at a high *j* of 1 A/cm^2^, mainly due to the rapid transport of sufficient H^+^ and CO_2_ in the bulk electrolyte. In contrast, in the electrolytes with pH of 4 and 7 (Supplementary Figs. 12b, c), the available CO_2_ concentrations began to decrease rapidly at a much lower *j* and recover to a higher level at further distances from the cathode than that at pH 1, implying a greater CO_2_ carbonation in an electrolyte with insufficient H^+^. Therefore, in order to pursue efficient CO_2_RR and high CO_2_ SPCE at high *j*, we sought to conduct CO_2_RR in strongly acidic electrolytes (pH 1), and attempted to explore, including the effect of CO_2_ flow rate, H^+^ and K^+^ concentrations on it.

To overcome the problem of (bi)carbonate formation and minimize the effect of CO_2_ carbonation under high *j*, we attempted to modulate the H^+^ concentration in the electrolyte (bulk pH) to neutralize the locally generated OH^−^, preventing the formation of (bi)carbonate. As shown in Fig. 3c, when *j* ≤ 0.5 A/cm^2^, the CO FE decreased with the pH decrease at the same *j*, whereas the H_2_ FE exhibited the opposite trend (Fig. 3d and Supplementary Fig. 13). Additionally, the onset *j* of CO_2_RR to CO increased gradually with the decrease of pH from 3 to 0.5 (Fig. 3c). This can be attributed to the fact that with the decrease in pH, the local H^+^ concentration of the electrode increases rapidly, and the HER is more likely to dominate^40,41^. Thus, a higher value of *j* is required to consume a substantial amount of H^+^ and modify the surface pH to make it more favorable for the kinetics of CO_2_RR. That is the shift of onset *j* for CO_2_RR strongly depends on bulk electrolyte pH, which is consistent with the simulation results and other reports (Fig. 3a)^22–26^. When the applied *j* was further increased from 0.5–2 A/cm^2^, all the CO FEs exceeded 95% in the 4–1 pH range, and CO FE exceeded 80% at pH 0.5 (Fig. 3e and Supplementary Fig. 14). Interestingly, when the *j* > 2 A/cm^2^, the CO FE increased as the pH decreases from 4 to 1 at the same *j*. This was irrespective of the decrease of all CO FE in the 4–0.5 pH range, which occurred as *j* further increased. Notably, at pH 0, almost no CO_2_RR was observed at any given *j* (Figs. 3e, f and Supplementary Figs. 13f, 14f). This is because HER always dominated when the surface pH could not be modulated due to the local H^+^ consumption rate being significantly lower than the mass transport rate of the bulk H^+^ in the extremely acidic electrolytes^22,23^.

**Fig. 4:**
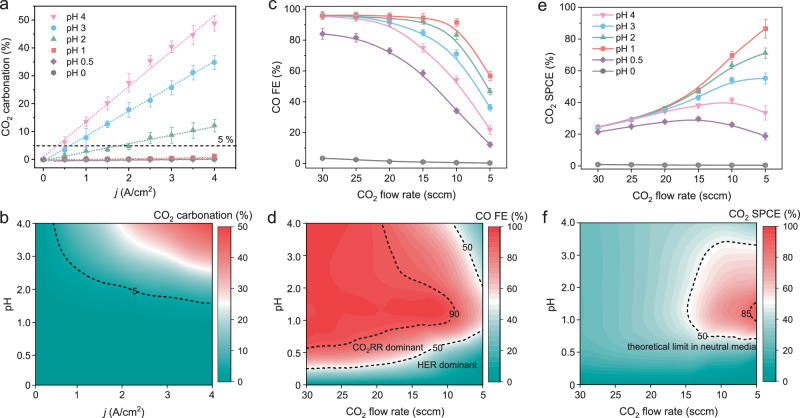
Effects of H^+^ concentration and CO_2_ flow rate on CO_2_RR. **a** CO_2_ carbonation percentage over CD-Ag HPE as a function of applied *j* measured in 3 M KCl + H_2_SO_4_ catholytes with different pH values. **b** pH−*j*-dependent mapping distribution of CO_2_ carbonation percentage over CD-Ag HPE. **c** CO FE and (**e**) CO_2_ SPCE over CD-Ag HPE as a function of input CO_2_ flow rate in 3 M KCl + H_2_SO_4_ catholytes with different pH values at a constant *j* of 2 A/cm^2^. The pH−CO_2_ flow rate-dependent mapping distribution of (**d**) CO FE and (**f**) CO_2_ SPCE over CD-Ag HPE at a constant *j* of 2 A/cm^2^. The error bars in (**a**), (**c**), (**e**) represent one standard deviation based on five independent tests.

Concurrently, the CO_2_ carbonation of different pH electrolytes at different *j* was further investigated (Fig. 4a and Supplementary Fig. 15). We discovered that in a near-neutral electrolyte (pH 4), the CO_2_ carbonation percentage increased rapidly from 6.2% to 48.9% as *j* increased from 0.5 to 4 A/cm^2^. However, CO_2_ carbonation decreased significantly as the pH of the electrolyte decreased. Accordingly, when pH ≤ 1, the CO_2_ carbonation was always < 5%, even when *j* reached 4 A/cm^2^ (Fig. 4a). To visualize the change in the trend of CO_2_ carbonation, the contour mapping distribution of CO_2_ carbonation on a pH−*j* plane (Fig. 4b) was plotted based on the date of Fig. 4a. It was clearly demonstrated that the CO_2_ carbonation increased rapidly in tandem with both the increase in *j* and the bulk pH (Fig. 4b). Correspondingly, the region with the greatest CO_2_ carbonation selectivity was located in the upper right of this map, indicating that the higher *j* as well as higher pH, the more serious CO_2_ carbonation. Based on the aforementioned findings, we reasoned that for an electrolyte with insufficient H^+^ concentration, the local OH^–^ was generated rapidly as *j* increased. Some fraction of the input CO_2_ would readily react with a large amount of locally generated OH^–^ to form a (bi)carbonate coating on the surface of the electrode resulting in a significant reduction of local available CO_2_ and CO_2_RR active sites^28,42^. As a result, when CO_2_RR at high *j*, local CO_2_ and CO_2_RR active sites of the electrode were insufficient, and HER was kinetically more favorable. However, an acidic electrolyte with sufficient H^+^ concentration can provide sufficient H^+^ to maintain a moderate local pH, effectively prevent CO_2_ carbonation, and ensure sufficient local CO_2_ for high-efficiency CO_2_RR at high *j* (Fig. 1).

### Effects of H^+^ concentration and CO_2_ flow rate on CO_2_RR

To further confirm this hypothesis, we reduced the flow rate of the input CO_2_ at a high *j* of 2 A/cm^2^ to make the effect of CO_2_ carbonation on CO_2_RR more apparent. As depicted in Fig. 4c, when the input CO_2_ flow rate was reduced from 30 to 25 sccm at pH 4, the H_2_ FE remained > 3%, and the CO FE remained virtually unchanged (> 97%). This is because a high flow rate of input CO_2_ could ensure relatively adequate local CO_2_ for high-efficiency CO_2_RR, even though ≈ 25% of CO_2_ was lost (Fig. 4a). However, as the input CO_2_ flow rate was further reduced from 25 to 5 sccm, the CO FE decreased rapidly (from 97% to 22%), while the H_2_ FE increased dramatically (Fig. 4c and Supplementary Fig. 16a). Thus, it can be inferred that under the dual influence of CO_2_ carbonation (Fig. 4a) and decreasing input CO_2_ flow rate, the local CO_2_ could not satisfy high-efficiency CO_2_RR at such a high *j*. In addition, the locally generated (bi)carbonate on the surface of the electrode may reduce the availability of CO_2_RR active sites^28,42^. Although the CO_2_ SPCE gradually increased by decreasing the CO_2_ flow rate to limit the availability of CO_2_ (Fig. 4e and Supplementary Fig. 16i), it was still < 50% of the theoretical limit value for neutral electrolytes^22,28^.

Then we reduced the input CO_2_ flow rate of CD-Ag HPE in different pH electrolytes and observed the effect on HER (Supplementary Fig. 16h) and CO_2_RR (Fig. 4c). In the pH range 1–4 when the CO_2_ flow rate was ≥ 20 sccm, the HER was effectively suppressed (Supplementary Fig. 16a–d), and the CO FE was always high (Fig. 4c; FE_CO_ > 90%). However, when the pH was < 1, an excessively high local H^+^ concentration increased H_2_ FE significantly (Supplementary Fig. 16e, f)^22,23^. Moreover, as the CO_2_ flow rate fell below 20 sccm, the CO FE decreased, and the H_2_ FE increased dramatically. However, at identical low input CO_2_ flow rates, CO FE had a volcano-like distribution in the 0–4 pH range. Consequently, at a high *j* of 2 A/cm^2^, the contour mapping distribution of CO FE on the pH−CO_2_ flow rate plane clearly showed that, at a low CO_2_ flow rate, the higher CO FE region was located at pH 1–2, in spite of CO FE is basically the same in pH 1–4 at high CO_2_ flow rate (Fig. 4d). For the distribution of CO_2_ SPCE (Fig. 4e), which was determined jointly by the *j*_CO_ and input CO_2_, the CO_2_ SPCE increased rapidly at pH 1 and 2 with a decrease in the input CO_2_ flow rate (from 20–5 sccm). Thus, when combined with the CO FE and input CO_2_ flow rate, the contour mapping distribution of CO_2_ SPCE on the pH−CO_2_ flow rate plane directly showed that the most CO_2_ SPCE selective area was located in the middle right of the map, where require both low input CO_2_ flow rate and low pH of 1–2 (Fig. 4f). In other words, at a CO_2_ flow rate of 5 sccm, the CO_2_ SPCE at a pH of 1–2 exceeded 80%, which is roughly double that at pH 4 (Fig. 4e). In addition, the *j*−CO_2_ flow rate-dependent mapping distribution of theoretical limit of CO FE based on CO_2_ carbonation in different pH electrolyte also showed a higher theoretical limit of CO FE could be achieved only in a strong acidic electrolyte (Supplementary Fig. 17).

These observations indicated that a high flow rate of CO_2_ can ensure high local CO_2_ for high-efficiency CO_2_RR but limit CO_2_ SPCE. However, in electrolytes with insufficient H^+^ concentration, the CO_2_ SPCE cannot be promoted effectively by merely reducing the input CO_2_. It is worth noting that the locally generated (bi)carbonate on the surface of the electrode not only causes a reduction of locally available CO_2_ but also covers the active sites for CO_2_RR, resulting in the collapse of CO FE at low input CO_2_ flow rate. When CO_2_RR was conducted in an acidic electrolyte, the proper H^+^ concentration in the electrolyte could effectively solve the problem of CO_2_ carbonation and ensure that the local CO_2_ concentration was sufficient despite a low input CO_2_ flow rate. Therefore, while CO_2_ availability is constrained, high-efficiency CO_2_RR can be maintained by reducing the input CO_2_, effectively promoting the CO_2_ SPCE.

### K^+^ effect on acidic CO_2_RR

**Fig. 5:**
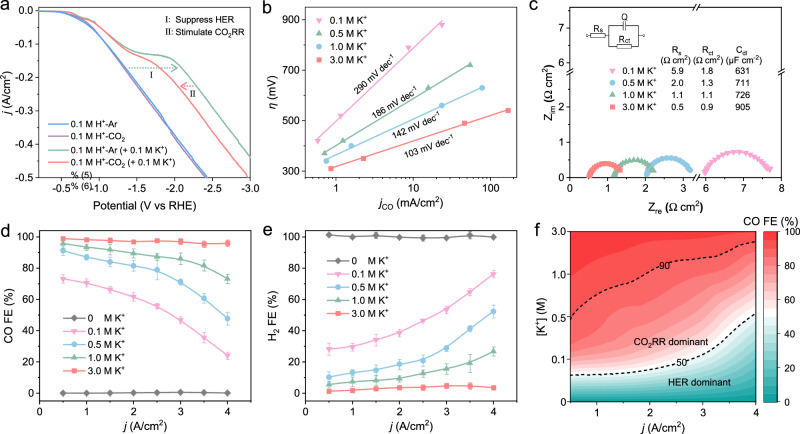
K^+^ effect on acidic CO_2_RR. **a** LSV curves of CD-Ag HPE in Ar-saturated or CO_2_-saturated pure H_2_SO_4_ or H_2_SO_4_ with 0.1 M K^+^ catholytes at pH 1. **b** Tafel slopes and **c** EIS Nyquist plots obtained in catholytes with different K^+^ concentrations at pH 1. **d** CO and (**e**) H_2_ FE over CD-Ag HPE as a function of applied *j* measured in H_2_SO_4_ catholytes with different K^+^ concentrations at pH 1 (input CO_2_ flow rate: 30 sccm). **f** *j* − K^+^ concentration-dependent mapping distribution of CO FE over CD-Ag HPE (input CO_2_ flow rate: 30 sccm). The error bars in (**d**), (**e**) represent one standard deviation based on five independent tests.

Given the essential role of alkali cation in the activation of CO_2_ and inhibition of HER^22,24,25^, we first performed linear voltammetry curve (LSV) analysis (Fig. 5a). In 0.05 M H_2_SO_4_ (pH 1) without any K^+^ electrolyte, the voltammetric properties of the CD-Ag HPE hardly changed regardless of the surrounding atmosphere (Ar or CO_2_), indicating that only HER occurs. However, HER activity was significantly suppressed by K^+^ presentation, concurrently, CO_2_RR occurred. This may be attributed to the fact that the presence of K^+^ in the acidic electrolyte shielded the electric field in the diffusion layer of the cathode and reduced the H^+^ concentration around the active site, which not only suppressed the HER, but also stimulated CO_2_ activation and conversion. (Supplementary Fig. 18a)^22,24,41^. Subsequently, we conducted Tafel analysis in 0.05 M H_2_SO_4_ + KCl catholytes at pH 1 but with different K^+^ concentrations (Fig. 5b). On the one hand, the CO Tafel slope was found to decrease with increasing K^+^ concentrations, reaching a minimum (104 mV dec^−1^) at 3 M K^+^. This result suggests that the rate-determining step (RDS) for CO formation comprised the adsorption of CO_2_, which could be altered with the K^+^ concentrations^22,43,44^. In other words, the activation energy barrier of the electron transfer over the electrode may be reduced in an electrolyte with a high K^+^ concentration, which is consistent with the faster initial one-electron transfer step required to form an adsorbed *COO^−^ intermediate. On the other hand, it can be seen that with the increase of K^+^ concentration, the changing trend of H_2_ Tafel slope value was opposite to that of CO. That is, the H_2_ Tafel slope value increased with increasing of K^+^ concentrations (Supplementary Figs. 19b, c). This is consistent with the results of LSV (Fig. 5a), indicating that the presence of K^+^ would suppress the HER. In addition, the total Tafel slope values of CO and H_2_ gradually decreased with increasing of K^+^ concentrations (Supplementary Fig. 19c), which was consistent with the results in Fig. 5c, where a faster electron transfer was verified by the lowest interfacial charge transfer resistance (*R*_ct_) of CD-Ag HPE in 3 M K^+^ (0.9 Ω cm^2^, Fig. 5c and Supplementary Table 8). In addition, the higher effective electric double layer capacitance (C_dl_) of CD-Ag HPE in acidic electrolyte with high K^+^ concentration, which was correlated with the electric field strength an enhanced electric field trend (Fig. 5c and Supplementary Table 8). These results are consistent with the hypothesis, that is the hydrated K^+^ physisorbed on the cathode in the acidic electrolyte modify the distribution of electric field in the double layer, which not only impedes HER by suppression of migration of H^+^, but also promotes CO_2_ reduction by stabilization of key intermediates (Supplementary Fig. 18)^22,24,41^.

We also examined the CO_2_RR product distribution in 0.05 M H_2_SO_4_ (pH 1) with varying K^+^ concentrations at different *j* (Fig. 5d, e and Supplementary Fig. 20). Even at a high *j* of 4 A/cm^2^, almost no CO could be detected in 0.05 M H_2_SO_4_ without K^+^ (Fig. 5d and Supplementary Fig. 20a), which was consistent with the LSV results (Fig. 5a). In addition, the HER selectivity (Fig. 5e) decreased as the K^+^ concentration increased, while the CO_2_RR selectivity (Fig. 5d) increased for all given values of *j*. Particularly, at a constant high *j* of 2 A/cm^2^, the CO FE increased steadily from 61.2% with 0.1 M K^+^ to 96.8% with 3 M K^+^. The contour mapping distribution of CO FE on the *j* − K^+^ concentration plane further directly showed that, CO_2_RR-dominated regions require the presence of high concentrations of K^+^, while obtaining high CO FE at high *j* is more dependent on high K^+^ concentrations (Fig. 5f). In addition, the porous micro/nanostructured Ag (Supplementary Fig. 21) was found to be conducive to increasing the local K^+^ concentration^22,45,46^, which can be attributed to the amplified electric field near the pore sites, thereby inhibiting HER and promoting the activity of CO_2_RR (Supplementary Fig. 22). Note that both controlled experiments and DFT calculations showed that, in an acidic electrolyte, the effect of anions on CO_2_RR reactivity was not significant; substitution of Br^–^, SO_4_^2–^ or PO_4_^3–^ for Cl^–^ showed product distribution similar to that of the Cl^–^ case (Supplementary Fig. 23), and the Gibbs free energy of *COOH and *H, the key intermediates from CO_2_RR to CO and HER, respectively, basically did not change whether there was Cl^-^ adsorption or not (Supplementary Figs. 24, 25).

### Effects of CO_2_ flow rate and *j* on acidic CO_2_RR

**Fig. 6:**
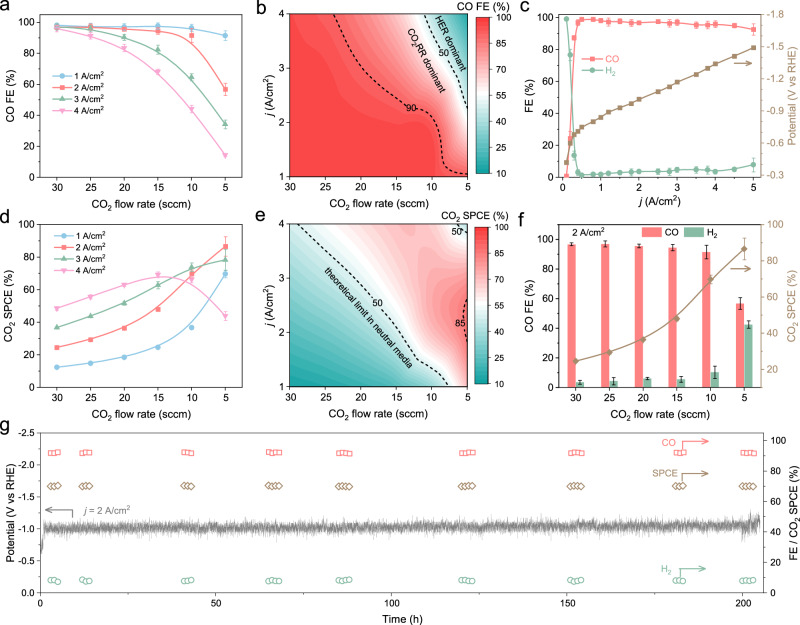
Effects of CO_2_ flow rate and *j* on acidic CO_2_RR. **a** CO FE and (**d**) CO_2_ SPCE over CD-Ag HPE as a function of input CO_2_ flow rate measured in 3 M KCl + 0.05 M H_2_SO_4_ catholytes (pH 1) at different applied *j*. The *j*–CO_2_ flow rate-dependent mapping distribution of (**b**) CO FE and (**e**) CO_2_ SPCE over CD-Ag HPE in 3 M KCl + 0.05 M H_2_SO_4_ catholytes (pH 1). **c** CO, H_2_ FE and potential over CD-Ag HPE as a function of applied *j* measured in 3 M KCl + 0.05 M H_2_SO_4_ catholytes (pH 1, input CO_2_ flow rate: 30 sccm). **f** CO, H_2_ FE and CO_2_ SPCE over CD-Ag HPE as a function of input CO_2_ flow rate measured in 3 M KCl + 0.05 M H_2_SO_4_ catholytes (pH 1) at a constant *j* of 2 A/cm^2^. **g** Long-term performance at a constant *j* of 2 A/cm^2^ in 3 M KCl + 0.05 M H_2_SO_4_ catholytes (pH 1, input CO_2_ flow rate: 10 sccm). The error bars in (**a**), (**c**), (**d**), (**f**) represent one standard deviation based on five independent tests.

To pursue a high CO_2_ SPCE under high CO_2_RR to CO activity, we analyzed the input CO_2_ flow rate effect on acidic CO_2_RR product distribution at various *j* (Fig. 6a and Supplementary Fig. 26). In strong acidic electrolytes, there is almost no loss of CO_2_ and CO_2_RR active sites because, in the absence of (bi)carbonate precipitation, the activation of high K^+^ concentration and the high input flow rate of CO_2_ can guarantee sufficient CO_2_ and CO_2_RR active sites locally on the catalyst surface for high*-*efficiency CO_2_RR. Consequently, the CO FE could maintain a high value (FE_CO_ > 95%) even when the *j* reached 4 A/cm^2^ (Fig. 6a and Supplementary Fig. 26). As the input flow rate decreased, the local CO_2_ supply became relatively insufficient, and as *j* increased, the CO FE began to decline rapidly. Thus, combined with the CO FE and *j*, the most *j*_CO_ selective region of the *j*–CO_2_ flow rate-dependent mapping distribution of CO FE is located in the top left corner, where the high flow rate of input CO_2_ could maintain the sufficient CO_2_ supply to achieve high CO FE at high *j* due to the unique structure of the HPE (Fig. 6b). These results indicate that a high input CO_2_ flow rate is necessary for CD-Ag HPE to achieve high-efficiency CO_2_RR to CO under high *j* in acidic electrolyte. The detailed performances of the CD-Ag HPE at high CO_2_ flow rate (30 sccm) revealed that the H_2_ FE remained < 5% as the *j* increased, up to 4.5 A/cm^2^, while the CO FE remained as high as 95.06%, yielding 4.28 A/cm^2^ *j*_CO_ at −1.41 V vs. RHE (Fig. 6c and Supplementary Fig. 27). The corresponding CO yield and CO energy efficiency were 80.83 mmol/(h cm^2^) and 48.25%, respectively (Supplementary Fig. 27 and Supplementary Table 2). Such performance stands out among recently reported prominent electrocatalysts for CO formation from CO_2_RR (Supplementary Fig. 28 and Supplementary Table 1).

Regarding CO_2_ SPCE, it is determined by both the *j*_CO_ and input CO_2_ flow rate. At a high input CO_2_ flow rate of > 20 sccm, the CO FE of CD-Ag HPE could remain above 80% even as *j* increased to 4 A/cm^2^ (Fig. 6a), whereas the CO_2_ SPCE increased only very slowly as *j* was increased (Fig. 6d, e). However, a moderate CO_2_ flow rate and a substantial CO FE at high *j* were more conducive to achieving a high CO_2_ SPCE. This trend was further visualized in the the *j*-CO_2_ flow rate-dependent mapping distribution of CO_2_ SPCE (Fig. 6e). The most CO_2_ SPCE selective region was located in the middle right of the map, where high CO_2_ SPCE of > 80% was only achieved when the *j* is around 2 A/cm^2^ and the flow rate is less than 10 sccm. Particularly, when the CO_2_ flow rate was decreased from 30–5 sccm at a constant high *j* of 2 A/cm^2^, the CO_2_ SPCE increased from 22% to 87% for CO_2_RR to CO, thereby exceeding the theoretical limit of 50% in neutral/alkaline systems. This was comparable to the record level of 90% at low *j* of < 0.2 A/cm^2^ in acidic systems (Fig. 6f and Supplementary Table 6)^28^.

Although highly challenging, the long-term operation of electrocatalysts under high current density is crucial for their practical applications^3,14^. Correspondingly, the *j* was fixed at 2 A/cm^2^, and the flow rate was set to 10 sccm in the acidic electrolyte stability test. During the 200-h continuous test, the CO FE remained consistently above 90%, while the corresponding CO_2_ SPCE fluctuated around 70% (Fig. 6g). In addition, the surface structures remained highly stable after the stability test (Supplementary Fig. 29), and the postreaction XRD and XPS further revealed the stable compositions of CD-Ag HPE after electrolysis (Supplementary Fig. 30 and Supplementary Table 10), which were responsible for the steady CO_2_ electroreduction performance. Thus, the overall CO_2_RR performance is well-placed among recently reported outstanding electrocatalysts for CO formation from CO_2_ reduction, including *j*, CO FE, CO yield (95% CO FE and 80.83 mmol/(h cm^2^) CO yield at high *j* of 4.5 A/cm^2^), CO_2_ SPCE and stability (87% CO_2_ SPCE and 200 h long-term test at *j* of 2 A/cm^2^) (Supplementary Figs. 26–31 and Supplementary Tables 1, 2, 6), demonstrating great potential for scalable application. And at the current density as high as 4.5 A/cm^2^, the overpotential of CD-Ag HPE was only 1.3 V, which was comparable to the high-current density electrocatalyst (Supplementary Table 1). In order to demonstrate the scalability for practical applications using hollow fiber penetration electrodes, single-, 2-, 5- and 10-tube arrays of CD-Ag HPE were further adopted and tested in a 2-electrode acidic system (pH = 1). All CD-Ag HPE array electrodes with different tube numbers showed highly similar FE distributions of CO and H_2_ at given high *j* range (Supplementary Figs. 32, 33). Thus, the *j*_CO_ over these CD-Ag HPE array electrodes also exhibited almost same rapidly growth trend with increasing *j*. Although CO FE and *j*_CO_ over the CD-Ag HPE array electrodes slightly decreased with increasing tube number and *j*, 10-tube CD-Ag HPE array still possessed over 90% of CO FE at high *j* of 4 A cm^−2^. These results implied the potential scalability for practical applications using hollow fiber penetration electrodes.

### Theoretical calculations

**Fig. 7:**
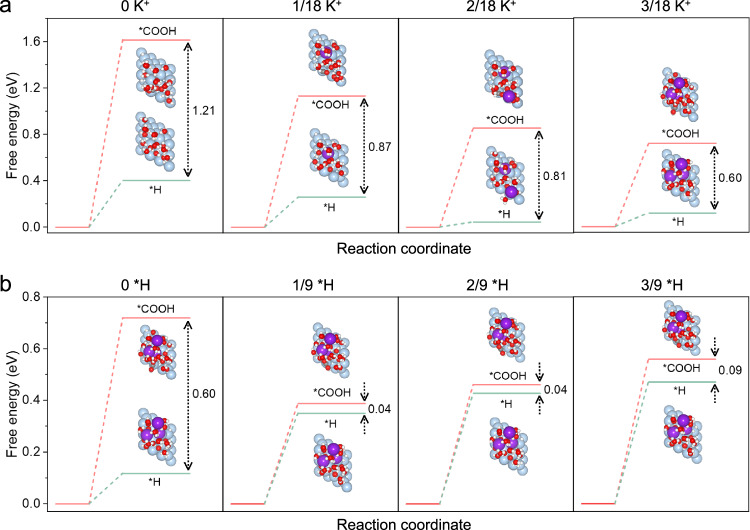
Theoretical calculations. The free energy for the formation of *COOH (G(*COOH)), *H (G(*H)), and their energy difference (G(*COOH)-G(*H)) on Ag (111) plane at various (**a**) K^+^ concentrations (1/18 per H_2_O molecule) and (**b**) *H coverages (1/9 per site). The light blue, purple, red, gray and white balls represent Ag, K, O, C and H, respectively.

The promoting mechanism of K^+^ and H^+^ in the aqueous microenvironment surrounding the Ag active site of the CD-Ag HPE was further simulated using DFT calculations. The formation of adsorbed COOH (*COOH) and H (*H) intermediates at the active site were thought to be the RDS for CO_2_RR and HER (Supplementary Figs. 34, 35)^46–48^, respectively. Thus, we first simulated an Ag (111) surface with a K^+^ concentration ranging from 0 to 3 (1/18 per H_2_O molecule), and compared the Gibbs free energy (G) change difference for the formation of *COOH and *H (Fig. 7a). Although both the G(*COOH) and G(*H) decreased with increasing K^+^ concentration, the G(*COOH–*H) also decreased rapidly (from 1.21 eV to 0.60 eV) with the increase of K^+^. This was consistent with the experimental findings, and the results indicated that an increase in K^+^ concentration could promote both CO_2_RR and HER, with CO_2_RR being the more dominant effect. On this basis, we further calculated the G for the formation of *COOH and *H at different *H coverages ranging from 0 to 3 (1/9 per site) (Fig. 7b). Correspondingly, we found volcano-shaped relationships between G(*COOH) and *H coverage, which is in stark contrast to the result that G(*H) increases as *H coverage increases. Consequently, the G(*COOH–*H) reached a minimum of 0.04 eV in the optimized structures with a high K^+^ concentration and moderate *H coverage (3/18 K^+^−1/9 *H and 3/18 K^+^−2/9 *H). In addition, we explored the effect of different hydrogen sources on a model of 3 K^+^ (1/18 / H_2_O molecule) (Supplementary Figs. 35–38). It was found that the values of G (*COOH), G (*H) and G (*COOH–*H) calculated by the H_3_O^+^ model were basically the same as those calculated by the *H model, and they were all much lower than the model values of H_2_O as hydrogen source (Supplementary Fig. 39). Taken together, these DFT results suggest that the coexistence of H^+^ and K^+^ could promote the CO_2_RR synergistically. A high K^+^ concentration and a moderate *H coverage are likely to facilitate *COOH formation for CO_2_RR over *H formation for HER, which is consistent with the experimentally observed high CO_2_RR to CO conversion in strongly acidic media with a high K^+^ concentration.

Combined with controlled experiments and theoretical studies, we demonstrated that the presence of K^+^ in the acidic electrolyte controlled the onset of the CO_2_RR, and that a moderate concentration of H^+^ effectively prevented the carbonation of CO_2_ and CO_2_RR active sites due to the precipitation of (bi)carbonate, ensuring sufficient CO_2_ and CO_2_RR active sites at the catalyst surface for high-efficiency CO_2_RR at ampere-level current density. Thus, by optimizing the K^+^ and H^+^ concentration and CO_2_ flow rate in a strong acidic electrolyte, a high CO FE > 95% at 4.5 A/cm^2^ and >200 h of stability testing at 2 A/cm^2^ are achieved. In addition, by limiting the availability of input CO_2_, the CO_2_ SPCE for CO_2_RR reached 87% at a high *j* of 2 A/cm^2^, demonstrating remarkable CO_2_ conversion capability. This study, therefore, provides a means for high-efficiency CO_2_ conversion in strong acid by modulating catalyst microenvironments, with great potential for practical application.

## Methods

### Chemicals and materials

Ag powder (99.9%, 50 nm) was purchased from Ningbo Jinlei Nano Materials Co., Ltd. Polyetherimide (PEI) was purchased from Saudi Basic Industries Corporation (SABIC). N-Methyl-2-pyrrolidone (NMP), sulfuric acid (H_2_SO_4_), potassium sulfate (K_2_SO_4_), potassium bicarbonate (KHCO_3_), potassium chloride (KCl) and potassium hydroxide (KOH) were purchased from Sinopharm Chemical Reagent Co., Ltd. Nafion 117 proton exchange membranes (PEMs) were purchased from DuPont. All chemicals were used as received without further purification. Electrolyte solutions were prepared using 18.2 MΩ H_2_O (ultrapure water, from Master-S30UVF water purification system).

### Catalyst preparation

Ag hollow fiber (Ag HF) was fabricated by a combined phase-inversion/sintering process (Supplementary Fig. 1)^37^. Briefly, commercially available polyetherimide (PEI, 24 g) was added to N-Methyl-2-pyrrolidone (NMP, 96 g), followed by ultrasonic treatment for 1 h to obtain a homogeneous and transparent solution. Then Ag powder (80 g) was added to the above solution. The as-obtained mixture was further treated by the planetary ball-milling (using 250 mL zirconia jar and ϕ5 mm zirconia balls) at 300 rpm for 24 h to form a uniform slurry. After cooling to room temperature, the slurry was vacuumed (1 mbar) for 5 h to remove bubbles and then to obtain a casting solution. Next, the casting solution was extruded through a spinning machine and shaped in a water bath via the phase-inversion process. After spinning, the as-formed tubes were kept in a water bath for 24 h to eliminate the solvent completely, followed by stretching and drying in ambient conditions with a humidity of ~28% for 48 h to obtain a green body. The green body was cut into appropriate lengths and then calcinated in an airflow (100 mL/min) at 600 °C (heating rate: 1 °C/min) for 6 h to remove PEI. After being naturally cooled to room temperature, the calcined green body was then reduced in a 5% H_2_ (argon balance) flow (100 mL/min) at 300 °C (heating rate: 1 °C/min) for 3 h to obtain Ag HF.

The Ag HF with an exposed length of 4 cm was stuck into a copper tube using conductive silver adhesive for electrical contact (see Fig. 2g for details), while the end of the Ag HF tube as well as the joint between the Ag HF and copper tube were sealed and covered with gas-tight and nonconductive epoxy. After drying at room temperature for 12 h, a working Ag HF penetration electrode (Ag HPE) was obtained with an exposed geometric area of 0.5 cm^2^ (S = πD_out_L = 3.14 × 400 × 10^−4 ^× 4 ≈ 0.5 cm^2^, where S is the electrode area, D_out_ is the outer diameter of hollow fiber, and L is the length of hollow fiber).

Ag_2_CO_3_-Ag HPE was synthesized from Ag HPE by electrochemical redox activation treatments. Typically, the Ag HPE was subjected to oxidation and reduction treatments on a Biologic VMP3 potentiostat using a three-electrode system in a gas-tight two-compartment electrolysis cell containing a Nafion 117 membrane as the separator, a KCl-saturated Ag/AgCl reference electrode and a platinum mesh (3 cm × 3 cm) counter electrode. The electrolyte solution was CO_2_-saturated 0.5 M KHCO_3_, and the CO_2_ flow rate was kept at 2 mL/min. Prior to the experiments, the electrolysis cell was vacuumized and then purged with CO_2_ for 30 min. The Ag HPE was electrochemically oxidized at a fixed potential of 2.0 V (vs. Ag/AgCl) for 4 min to obtain Ag_2_CO_3_-Ag HPE. Subsequently, the Ag_2_CO_3_-Ag HPE was reduced at a fixed potential of −0.50 V (vs. Ag/AgCl) for 10 min to obtain CD-Ag HPE. The CD-Ag HPE possessed the same exposed geometric area of 0.5 cm^2^ (S=πD_out_L = 3.14 × 400 × 10^−4 ^× 4 = 0.5 cm^2^). For the 10-tube CD-Ag HPE array electrode, the exposure geometric area was 5 cm^2^ (S = nπD_out_L = 10 × 3.14 × 400 × 10^−4 ^× 4 = 5 cm^2^, where n is the number of hollow fiber tubes). The electrochemical oxidation reaction and reduction reaction obeyed Eqs. (1) and (2), respectively.

1 $$2{Ag}+2{H}_{2}O+{HC}{O}_{3}^{-}\to A{g}_{2}C{O}_{3}+6{e}^{-}+{O}_{2}\uparrow+5{H}^{+}$$

2 $$A{g}_{2}C{O}_{3}+5{H}^{+}+6{e}^{-}\to 2{Ag}+{HC}{O}_{3}^{-}+{2H}_{2}\uparrow$$

In addition, the OD-Ag HPE was also treated in the Ar-saturated 0.5 M KOH with the same electrochemical redox activation treatments as that of CD-Ag HPE. That is, the Ag HPE that underwent 60 s of oxidation and 600 s of reduction in Ar-saturated 0.5 M KOH electrolyte solution, respectively.

### Material characterization

The cross-section and surface morphologies of samples were observed via scanning electron microscopy (SEM) with a SUPRRATM 55 microscope using an accelerating voltage of 5.0 kV. Transmission electron microscopy (TEM) investigations were conducted with a JEM-ARM300F microscope operated at 300 kV. X-ray diffraction (XRD) measurements were performed on a Rigaku Ultima 4 X-ray diffractometer using a Cu Kα radiation source (λ = 1.54056 Å) at 40 kV and 40 mA. X-ray photoelectron spectroscopy (XPS) tests were conducted using a Quantum 2000 Scanning ESCA Microprobe instrument with a monochromatic Al Kα source (1486.6 eV). The binding energies in all XPS spectra were calibrated according to the C 1 s peak (284.8 eV).

### Electrochemical measurements

All electrochemical measurements were performed using a Biologic VMP3 potentiostat in a two-compartment electrolysis cell at an ambient temperature and pressure. The electrolysis cell comprises two symmetrical compartments made of quartz glass with an inner height of 5.0 cm, an inner length of 5.0 cm and an inner width of 1.5 cm (Fig. 2g, h). The cathodic and anodic compartments were separated by a Nafion 117 membrane, and the electrolysis cell was equipped with a KCl-saturated Ag/AgCl reference electrode in the cathodic compartment and a platinum mesh (3 cm × 3 cm). CO_2_-saturated H_2_SO_4_ containing various concentrations of KCl or different pH was used as catholyte, and 0.5 M K_2_SO_4_ + 0.05 M H_2_SO_4_ aqueous solution was used as anolyte. Prior to the experiments, the electrolysis cell was vacuumed, and then CO_2_ was continuously delivered into the cathodic compartment at a constant rate of 30 mL/min for 30 min. During all of the electrochemical measurements, the CO_2_ flow rate was fixed at 30 mL/min. Note that the input CO_2_ flow rate was fixed at 30 mL/min during all electrochemical measurements, but the exhaust flow rate was not 30 mL/min at all due to the hydrogen evolution and CO_2_ consumption. Thus, the outlet flow rate of the electrolysis cell was measured by an independent Alicat® mass flowmeter (Fig. 2i and Supplementary Table 4). The cathodic electrolyte was 3.0 KCl + 0.05 M H_2_SO_4_, the anodic electrolyte was 0.5 M K_2_SO_4_ + 0.05 M H_2_SO_4_, unless otherwise stated.

For electrochemical characterizations, the electrochemical impedance spectroscopy (EIS) measurements were performed at −0.2 A/cm^2^ with a voltage amplitude of 50 mV, and the frequency limits were typically set in the range from 50 Hz to 500 kHz. The effective double-layer capacitance (Cdl) was obtained from the constant phase element (CPE) parameters and the two resistances using the Brug formula^37,49^:

3 $${C}_{{dl}}={T}^{\frac{1}{P}}\left({\frac{1}{{R}_{s}}}+{\frac{1}{{R}_{{ct}}}}\right)^{\frac{P-1}{P}}$$

Where R_s_ is the solution resistance, R_ct_ is the charge transfer resistance, T is CPE constant and P is CPE exponent. For the CO_2_ electroreduction tests, a Biologic VMP3 potentiostat was used for small current situations (≤ 400 mA), while the Biologic VMP3 potentiostat was connected to a VMP3 booster chassis with a 10 A current option to be used under large current situations (≥ 400 mA). The CO_2_RR performance of the CD-Ag HPE array electrodes with different tube numbers in the 2-electrode acidic system (pH = 1) was performed using an ANS6050D (50 V, 50 A) direct-current source from ANS Power Co., Ltd. with constant current control applied. In addition, both the anolyte and catholyte were cycled in anodic and cathodic compartments with a fixed flow rate of 50 mL/min by using two identical peristaltic pumps (JIHPUMP BT-50EA 153YX). For the long-term performance test of CO_2_ electroreduction, the current density was fixed at −2 A/cm^2^ in CO_2_-saturated 3 M KCl + 0.05 M H_2_SO_4_ catholyte, the exhaust from the cathodic compartment was measured by online GC during the entire 200-hour test.

All the current in the main text and supplementary materials were geometrically normalized to the electrode area. All the applied potentials were recorded against the KCl-saturated Ag/AgCl reference electrode and then converted to those versus the reversible hydrogen electrode (RHE) with iR corrections using the following equation:

4 $$E\left({{{\rm{vs}}}}.\, {{{\rm{RHE}}}}\right)=E\left({{{\rm{vs}}}}.\, {{{\rm{Ag}}}}/{{{\rm{AgCl}}}}\right)+0.197V+0.0591V\times {{{\rm{pH}}}}+0.85i{R}_{s}$$

Where E (vs. Ag/AgCl) is the applied potential, pH indicates the H^+^ concentrations of the electrolyte solutions (Supplementary Table 7), i is the current density at each applied potential, and R_s_ is the solution resistance obtained via EIS measurements (Supplementary Table 8). In order to avoid the overcorrected potentials, 85% iR correction was applied as the previous reports^17,48^. All applied potentials in the main text and Supplementary Information are referred to as RHE, unless otherwise stated. Note that the XY data at different pH values of Figs. 4a, c, e were first converted into XYZ data by the origin® software, to obtain the corresponding contour maps of Figs. 4b, d, f, respectively. Moreover, the XY data at different K^+^ concentrations of Fig. 5d in manuscript were first converted into XYZ data in origin® software, to obtain the corresponding contour map of Fig. 5f. In addition, the XY data at different current densities of Figs. 6a, d were first converted into XYZ data by the origin® software, to obtain the corresponding contour maps of Fig. 6b, e, respectively. Taking Fig. 4b for example, the X represents current density of Fig. 4a, Y represents pH of Fig. 4a, and Z represents percentage loss of CO_2_ of Fig. 4a, which were then standard smoothened and transformed back into a virtual matrix. The as-obtained virtual matrix was further presented in the form of a contour map, i.e., Fig. 4b, in consistency with the previous reports^28,50^.

### Product quantifications

Gas-phase products from the cathodic compartment were directly vented into a gas chromatograph (GC-2014, Shimadzu) equipped with a Shincarbon ST80/100 column and a Porapak-Q80/100 column using a flame ionization detector (FID) and a thermal conductivity detector (TCD) during the electroreduction tests and analyzed online. FID was used for CO quantification (as well as CH_4_, C_2_H_4_ and C_2_H_6_), while TCD was used for H_2_ and CO quantification. In addition, the concentration of unreacted CO_2_ in the outlet gas was analyzed with an independent Agilent 7890B gas chromatograph (Supplementary Fig. 9). A thermal conductivity detector (TCD) with a carbon molecular sieves column (TDX-1) was used for CO_2_ quantification. All faradaic efficiencies reported were based on at least three different GC runs, and the error bars of all figures in this work were based on the standard deviations of at least five independent electrochemical tests, unless otherwise stated. High-purity argon (99.999%) was used as the GC carrier gas. In all the CO_2_ electrolysis tests, only H_2_ and CO were the gas-phase products, and their faradaic efficiencies were calculated as follows:

5 $${FE}=\frac{{C}_{{product}}\times {10}^{-6}\times {v}_{{outlet}}\times {10}^{-3}\times t\times n\times F}{{V}_{m}\times Q}\times 100\%$$

where C_product_ is the concentration of the gas-phase products (ppm), ν_outlet_ is the outlet flow rate of the electrolysis cell. Note that the exhaust flow rate of the electrolysis cell was not equal to the input CO_2_ flow rate at all due to the hydrogen evolution and CO_2_ consumption. Thus, the outlet flow rate of electrolysis cell was measured by an independent Alicat® mass flowmeter (Fig. 2i). The actual measured outlet flow rate was higher than the inlet flow rate due to HER occurrence (Supplementary Table 3 and Fig. 5f). Thus the actual exhaust outlet flow rates (corrected v_outlet_) were measured by the Alicat® mass flowmeter (Fig. 2i), which possessed a full scale of 50 sccm with the accuracy ± (0.8% of Reading + 0.2% of Full Scale), as shown in the note d of Supplementary Table 3. That is the experiment errors caused by the Alicat® mass flowmeter could be negligible under the CO_2_ electroreduction conditions. In addition, the Alicat® mass flowmeter results were calibrated using a mixture (including CO, H_2_, CO_2_ and water vapor compositions at room temperature) that approximates the actual outlet gas composition (Supplementary Table 4). t is the reaction time, n is the number of transferred electrons for producing CO or H_2_, F is the Faraday constant, V_m_ is the gas mole volume, and Q is the total quantity of the electric charge. The CO formation rate was calculated using the following equation:

6 $${CO\; formation\; rate}=\frac{Q\times {{FE}}_{{CO}}}{F\times n\times t\times S}$$

Where S is the geometric area of the electrode (cm^2^).

The *j*_CO,limit(gas)_ is the theoretical limit of CO partial current density with all gas-phase CO_2_ molecules input into the electrolysis cell were reduced to CO products. The theoretical limits of CO product partial current density, i.e., *j*_CO,lim(gas)_ were calculated using the following equation^37,38^:

7 $${j}_{{CO},{lim}({gas})}=\frac{{nF}}{S}\frac{{v}_{{{CO}}_{2}}}{{V}_{m}}$$

So, the theoretical limits of CO FE, i.e., FE_CO,lim(gas)_ were calculated by the following equation:

8 $${{FE}}_{{CO},{lim}({gas})}=\frac{{j}_{{CO},{lim}({gas})}}{{j}_{{total}}}\times 100\%$$

The CO_2_ SPCE, the CO_2_ carbonation and unreacted CO_2_ were calculated as follows:

9 $${{{\rm{SPCE}}}}=\frac{{{produced\; CO}}}{{{Input}}\, {{{CO}}}_{2}}\times 100\%$$

10 $${{CO}}_{2}\, {carbonation}=\frac{{Input}\, {{CO}}_{2}-{Unreacted}\, {{CO}}_{2}-{Converted}\, {{CO}}_{2}\,}{{Input}\, {{CO}}_{2}}\times 100\%$$

11 $${Unreacted}\, {{CO}}_{2}={V}_{{outlet}}-{Prodeced\; CO}-{Prodeced}\, {H}_{2}$$

12 $${Converted}\, {{CO}}_{2}={Prodeced\; CO}$$

Where the input CO_2_ (V_input_) of electrolysis cell was controlled by a Alicat® mass flow controller, and the outlet flow rate (V_outlet_) of electrolysis cell was measured by an independent Alicat® mass flowmeter. The actual outlet gas of electrolysis cell contains unreacted CO_2_, produced CO (from CO_2_RR) and produced H_2_ (from the HER). The produced CO and H_2_ at given current density could be quantified by online GC. Furthermore, to verify our calculation of unreacted CO_2_, the concentration of unreacted CO_2_ in the outlet gas was measured by an independent online GC (Fig. 2i).

By assuming that the overpotential of oxygen evolution reaction on the anode side is zero, the cathodic energy efficiency for CO was calculated as follows^12^:

13 $${{EE}}_{{CO}}=\frac{(1.23+(-{E}_{{CO}}))\times {{FE}}_{{CO}}}{1.23+(-E)}$$

Where E_CO_ is −0.11 V (vs. RHE); 1.23 V is the thermodynamic potential for water oxidation in the anode side.

Possible liquid-phase products from the cathodic compartment after CO_2_ electrolysis for 1 h were analyzed using another off-line GC-2014 (Shimadzu) equipped with a headspace injector and an OVI-G43 capillary column (Supelco, USA). No liquid-phase product was detected by the off-line GC. The post-reaction catholyte solution was also analyzed by a 600 MHz NMR spectrometer (Bruker) for possible liquid-phase products (especially formate and acetate). After an hour of electrolysis, an aliquot of catholyte solution (0.5 mL) was mixed with 0.1 mL (CH_3_)_3_Si(CH_2_)_3_SO_3_Na (DSS) (6 mM) and 0.1 mL D_2_O for use as internal standards. No liquid-phase product was detected by ^1^H NMR (Supplementary Figs. 6–9).

### COMSOL multiphysics simulations

A reaction-diffusion model (Supplementary Fig. 10) was used to simulate the local pH and CO_2_ concentration using COMSOL Multiphysics software in a typical 50 μm diffusion layer^22,23,25^. All the interactions between species in the electrolyte (CO_2_, HCO_3_^-^, CO_3_^2-^, SO_4_^2-^, K^+^, Cl^-^, OH^-^, H^+^ and H_2_O) were considered (Supplementary Table 11). Specifically, one end of the one-dimensional simulation area is set as the working electrode surface, and the other side is set as the bulk concentration to describe the bulk electrolyte. We used Henry’s law to calculate the CO_2_ concentration, assuming that the CO_2_ fugacity is 1 bar.

14 $${C}_{{CO}2,{aq}}^{0}={K}_{H}^{0}{C}_{{CO}2,{gas}}^{0}$$

${{{{\rm{K}}}}}_{{{{\rm{H}}}}}^{0}$ is the Henry’s constant, which can be calculated by using the equation below, where T is the temperature.

15 $${{\mathrm{ln}}}{K}_{H}^{0}=93.457\times \frac{100}{T}-60.2409+23.3585\times {{\mathrm{ln}}}\frac{T}{100}$$

Due to the high concentration of the ions, the saturated concentration of CO_2_ in an electrolyte is corrected using the following equations.

16 $$\log \frac{{C}_{{CO}2,{aq}}^{0}}{{C}_{{CO}2,{aq}}}={K}_{s}{C}_{s}$$

17 $${K}_{s}\,=\,\sum ({h}_{{ion}}\,+\,{h}_{G})$$

18 $${h}_{G}={h}_{G,0}+{h}_{T}(T-298.15)$$

*C*_*s*_ is the molar concentration and *K*_*s*_ is the Sechenov’s constant.

We considered the following homogeneous and heterogenous reactions in our model, which are based on the previously published works^22,23,25^. The heterogenous reactions take place in the electrolyte as follow:

19 $$2{H}_{2}O+2{e}^{-}\to {H}_{2}+2O{H}^{-}$$

20 $${{{\rm{C}}}}{O}_{2}+{H}_{2}O+2{e}^{-}\to {{{\rm{CO}}}}+2O{H}^{-}$$

In this work, two sets of Butler-Volmer boundary conditions (CO_2_RR and HER) were set to represent the two main sets of reactions on electrode surface, the kinetic parameters sets for these two reactions were the Tafel slopes of CO (103 mV dec^−1^) and H_2_ (56 mV dec^−1^), respectively, and the FEs of CO and H_2_ in the model were calculated and simulated based on these parameters.

Over the whole domain, the following homogenous reactions occur:

21 $${{{\rm{C}}}}{O}_{2}+{H}_{2}O\, \rightleftharpoons \, {H}^{+}+{HC}{O}_{3}^{-}$$

22 $${HC}{O}_{3}^{-}\, \rightleftharpoons \, {H}^{+}+C{O}_{3}^{2-}$$

23 $${{{\rm{C}}}}{O}_{2}+O{H}^{-}\, \rightleftharpoons \, {HC}{O}_{3}^{-}$$

24 $${HC}{O}_{3}^{-}+O{H}^{-}\, \rightleftharpoons \, C{O}_{3}^{2-}+{H}_{2}O$$

25 $${H}_{2}O\rightleftharpoons {H}^{+}+O{H}^{-}$$

The bulk concentrations and pH values were measured experimentally and implemented in the model. The thickness of the diffusion layer was assumed to be 50 μm. The electrode surface undergoes a reduction reaction, its current characteristics follow the Bulter-Volmer equation and the Nernst equation, its potential characteristics follow the Nernst equation, the concentration of the opposite body phase is set to the corresponding initial concentration, and the ion migration in the simulation area follows the Nernst Planck equation. The solution process is based on the MUMPS (multiple massively parallel spark direct solver) steady-state solver, and the relative tolerance and residual factor are set to 1E^−8^ and 1, respectively, eight layers of boundary layer subdivision are set on the simulated electrode surface to ensure the accuracy of the simulation results. The pH and CO_2_ concentration distribution near the electrode surface was calculated by solving the operating current from 0–1000 mA/cm^2^ at bulk pH of 1, 4, and 7, respectively.

The electrode surface reaction follows the BV equation:

26 $${i}_{{loc}}={i}_{0}\left(\exp \left(\frac{{\alpha }_{a}{Fn}\eta }{{RT}}\right)-\exp \left(\frac{-{\alpha }_{c}{Fn}\eta }{{RT}}\right)\right)$$

Where i_loc_ is the local current density at the electrode/electrolyte interface, i_0_ is the exchange current density, ${{{{\rm{\alpha }}}}}_{{{{\rm{c}}}}}$ and ${{{{\rm{\alpha }}}}}_{{{{\rm{a}}}}}$ is the cathodic and anodic charge transfer coefficients, *η* is the activation overpotential.

The balance potential follows the Nernst equation:

27 $${E}_{{eq}}=-\frac{\triangle G}{{nF}}$$

28 $${E}_{{eq}}={E}_{{eq},{ref}}-\frac{{RT}}{{nF}}{{\mathrm{ln}}}{\prod}_{i}{\left(\frac{{a}_{i}}{{a}_{i,{ref}}}\right)}^{{\nu }_{i}}$$

Where E_eq_ is the electrode potential, ΔG is the Gibbs free energy, E_eq,ref_ is the standard electrode potential, ${{{{\rm{a}}}}}_{{{{\rm{i}}}}}$ is the (electrode reactive ion concentration),$\,{{{{\rm{a}}}}}_{{{{\rm{i}}}},{{{\rm{ref}}}}}$ is the (standard electrode reactive ion concentration), v_i_ is the reaction stoichiometric number.

The transfer of dilute substances follows Fick's law:

29 $${N}_{i}={J}_{i}=-{D}_{i}\nabla {c}_{i}$$

30 $$\frac{\partial {c}_{i}}{\partial t}+\nabla {N}_{i}={R}_{i,{tot}}$$

Where J_i_ is the ion flux, D_i_ is the diffusion coefficient (D_Li_ = 1 × 10^−7^ m^2^s^−1^), c_i_ is the concentration of ion, ∇ci is concentration gradient.

The transportation of electricity follows the Nernst Planck relationship:

31 $${N}_{i}=-{D}_{i}\nabla {c}_{i}-{z}_{i}{u}_{m,i}F{c}_{i}\nabla {{{\varnothing }}}_{l}={J}_{i}+u{c}_{i}$$

Where z_i_ is the transfer number (z_Li_ = 1), u_m,i_ is the electric mobility coefficient, *ϕ* is the electrolyte potential.

### Computational theoretical calculations

The present first principle DFT calculations are performed by Vienna Ab initio Simulation Package with the projector augmented wave method^51^. The exchange function is treated using the generalized gradient approximation of Perdew-Burke-Emzerh functional^52^. For Kohn–Sham wave functions, the cutoff energy of the corresponding plane-wave basis set was set to 450 eV. The K points meshing was obtained from the Monkhorst-Pack scheme^53^. Grimme’s DFT-D3 methodology was used to describe the dispersion interactions. A vacuum width of of 15 Å along the Z axis was created to ensure negligible interaction. The force convergence criterion was set to 0.02 eV/Å and energy convergence criterion was 10^−4^ eV. To fully consider the solvation effect, 18 explicit water molecules were optimized, and a local minimum via the hydrogen bond network was formed. The Gibbs free energy change (ΔG) of each step is calculated using the following formula:

32 $$\triangle G\,=\,\triangle E\,+\,\triangle {ZPE}-\,T\triangle S$$

where ΔE is the electronic energy difference directly obtained from DFT calculations, ΔZPE is the zero point energy difference, T is the room temperature (298.15 K) and ΔS is the entropy change.

### Supplementary information

Supplementary Information

Peer Review File

### Source data

Source Data

## Data Availability

The data supporting the findings of the study are available within the paper and its Supplementary Information. Source data are provided in this paper.

## References

[1] Davis, S. J., Caldeira, K. & Matthews, H. D. Future CO_2_ emissions and climate change from existing energy infrastructure. *Science* **329**, 1330–1333 (2010). 20829483 10.1126/science.1188566

[2] Chu, S. & Majumdar, A. Opportunities and challenges for a sustainable energy future. *Nature* **488**, 294–303 (2012). 22895334 10.1038/nature11475

[3] De Luna, P. et al. What would it take for renewably powered electrosynthesis to displace petrochemical processes? *Science* **364**, eaav3506 (2019). 31023896 10.1126/science.aav3506

[4] Fang, M., Xu, L., Zhang, H., Zhu, Y. & Wong, W.-Y. Metalloporphyrin-linked mercurated graphynes for ultrastable CO_2_ Electroreduction to CO with nearly 100% selectivity at a current density of 1.2 A cm^–2^. *J. Am. Chem. Soc.* **144**, 15143–15154 (2022). 35947444 10.1021/jacs.2c05059

[5] Huang, J.-R. et al. Single-product faradaic efficiency for electrocatalytic of CO_2_ to CO at current density larger than 1.2 A cm^−2^ in neutral aqueous solution by a single-atom nanozyme. *Angew. Chem. Int. Ed.* **134**, e202210985 (2022). 10.1002/ange.202210985 36068177

[6] Li, Y. et al. Atomically dispersed single Ni site catalysts for high-efficiency CO_2_ electroreduction at industrial-level current densities. *Energy Environ. Sci.* **15**, 2108–2119 (2022). 10.1039/D2EE00318J

[7] Yan, S. et al. Electron localization and lattice strain induced by surface lithium doping enable ampere-level electrosynthesis of formate from CO_2_. *Angew. Chem. Int. Ed.* **60**, 25741–25745 (2021). 10.1002/anie.202111351 34617366

[8] Wen, G. et al. Continuous CO_2_ electrolysis using a CO_2_ exsolution-induced flow cell. *Nat. Energy* **7**, 978–988 (2022). 10.1038/s41560-022-01130-6

[9] Ma, W. C. et al. Electrocatalytic reduction of CO_2_ to ethylene and ethanol through hydrogen-assisted C-C coupling over fluorine-modified copper. *Nat. Catal.* **3**, 478–487 (2020). 10.1038/s41929-020-0450-0

[10] de Arquer, F. P. G. et al. CO_2_ electrolysis to multicarbon products at activities greater than 1 A cm^-2^. *Science* **367**, 661–666 (2020). 32029623 10.1126/science.aay4217 PMC13531662

[11] Möller, T. et al. Efficient CO_2_ to CO electrolysis on solid Ni–N–C catalysts at industrial current densities. *Energy Environ. Sci.* **12**, 640–647 (2019). 10.1039/C8EE02662A

[12] Dinh, C. T. et al. CO_2_ electroreduction to ethylene via hydroxide-mediated copper catalysis at an abrupt interface. *Science* **360**, 783–787 (2018). 29773749 10.1126/science.aas9100

[13] Zhang, X. et al. Selective and high current CO_2_ electro-reduction to multicarbon products in near-neutral KCl electrolytes. *J. Am. Chem. Soc.* **143**, 3245–3255 (2021). 33617245 10.1021/jacs.0c13427

[14] Ma, M. et al. Insights into the carbon balance for CO_2_ electroreduction on Cu using gas diffusion electrode reactor designs. *Energy Environ. Sci.* **13**, 977–985 (2020). 10.1039/D0EE00047G PMC8163407 34123139

[15] Xu, Y. et al. A microchanneled solid electrolyte for carbon-efficient CO_2_ electrolysis. *Joule* **6**, 1333–1343 (2022). 10.1016/j.joule.2022.04.023

[16] Rabinowitz, J. A. & Kanan, M. W. The future of low-temperature carbon dioxide electrolysis depends on solving one basic problem. *Nat. Commun.* **11**, 5231 (2020). 33067444 10.1038/s41467-020-19135-8 PMC7567821

[17] Zhao, Y. et al. Industrial-current-density CO_2_-to-C_2+_ electroreduction by anti-swelling anion-exchange ionomer-modified oxide-derived Cu nanosheets. *J. Am. Chem. Soc.* **144**, 10446–10454 (2022). 35640069 10.1021/jacs.2c02594

[18] Xie, K. et al. Eliminating the need for anodic gas separation in CO_2_ electroreduction systems via liquid-to-liquid anodic upgrading. *Nat. Commun.* **13**, 3070 (2022). 35654799 10.1038/s41467-022-30677-x PMC9163163

[19] Ozden, A. et al. Carbon-efficient carbon dioxide electrolysers. *Nat. Sustain.* **5**, 563–573 (2022). 10.1038/s41893-022-00879-8

[20] Shin, H., Hansen, K. U. & Jiao, F. Techno-economic assessment of low-temperature carbon dioxide electrolysis. *Nat. Sustain.* **4**, 911–919 (2021). 10.1038/s41893-021-00739-x

[21] Burdyny, T. & Smith, W. A. CO_2_ reduction on gas-diffusion electrodes and why catalytic performance must be assessed at commercially-relevant conditions. *Energy Environ. Sci.* **12**, 1442–1453 (2019). 10.1039/C8EE03134G

[22] Huang, J. E. et al. CO_2_ electrolysis to multicarbon products in strong acid. *Science* **372**, 1074–1078 (2021). 34083485 10.1126/science.abg6582

[23] Xie, Y. et al. High carbon utilization in CO_2_ reduction to multi-carbon products in acidic media. *Nat. Catal.* **5**, 564–570 (2022). 10.1038/s41929-022-00788-1

[24] Gu, J. et al. Modulating electric field distribution by alkali cations for CO_2_ electroreduction in strongly acidic medium. *Nat. Catal.* **5**, 268–276 (2022). 10.1038/s41929-022-00761-y

[25] Qiao, Y. et al. Engineering the local microenvironment over Bi nanosheets for highly selective electrocatalytic conversion of CO_2_ to HCOOH in Strong Acid. *ACS Catal.* **12**, 2357–2364 (2022). 10.1021/acscatal.1c05135

[26] Löffelholz, M., Osiewacz, J., Weseler, L. & Turek, T. Enhancing carbon efficiency in electrochemical CO_2_ reduction at silver gas diffusion electrodes-the effect of acidic electrolytes explained via TFFA modeling. *J. Electrochem. Soc.* **170**, 123502 (2023). 10.1149/1945-7111/ad0eba

[27] Li, H. et al. Tailoring acidic microenvironments for carbon-efficient CO_2_ electrolysis over Ni-N-C catalyst in a membrane electrode assembly electrolyzer. *Energy Environ. Sci.* **16**, 1502–1510 (2023). 10.1039/D2EE03482D

[28] Pan, B. et al. Close to 90% single-pass conversion efficiency for CO_2_ electroreduction in an acid-fed membrane electrode assembly. *ACS Energy Lett.* **7**, 4224–4231 (2022). 10.1021/acsenergylett.2c02292

[29] Bondue, C. J., Graf, M., Goyal, A. & Koper, M. T. M. Suppression of hydrogen evolution in acidic electrolytes by electrochemical CO_2_ reduction. *J. Am. Chem. Soc.* **143**, 279–285 (2021). 33356205 10.1021/jacs.0c10397 PMC7809687

[30] Zhang, Y.-J., Sethuraman, V., Michalsky, R. & Peterson, A. A. Competition between CO_2_ reduction and H_2_ evolution on transition-metal electrocatalysts. *ACS Catal.* **4**, 3742–3748 (2014). 10.1021/cs5012298

[31] Cave, E. R. et al. Trends in the catalytic activity of hydrogen evolution during CO_2_ electroreduction on transition metals. *ACS Catal.* **8**, 3035–3040 (2018). 10.1021/acscatal.7b03807

[32] Yan, Z. F., Hitt, J. L., Zeng, Z. C., Hickner, M. A. & Mallouk, T. E. Improving the efficiency of CO_2_ electrolysis by using a bipolar membrane with a weak-acid cation exchange layer. *Nat. Chem.* **13**, 33–40 (2021). 33288894 10.1038/s41557-020-00602-0

[33] Liu, Z. K. et al. Acidic electrocatalytic CO_2_ reduction using space-confined nanoreactors. *ACS Appl. Mater. Interfaces* **14**, 7900–7908 (2022). 35107020 10.1021/acsami.1c21242

[34] Kas, R. et al. Three-dimensional porous hollow fibre copper electrodes for efficient and high-rate electrochemical carbon dioxide reduction. *Nat. Commun.* **7**, 10748 (2016). 26888578 10.1038/ncomms10748 PMC4759634

[35] Rabiee, H. et al. Shape-tuned electrodeposition of bismuth-based nanosheets on flow-through hollow fiber gas diffusion electrode for high-efficiency CO_2_ reduction to formate. *Appl. Catal. B-Environ.* **286**, 119945 (2021). 10.1016/j.apcatb.2021.119945

[36] Li, S. J. et al. Chloride ion adsorption enables ampere-level CO_2_ electroreduction over silver hollow fiber. *Angew. Chem. Int. Ed.* **61**, e202210432 (2022). 10.1002/anie.202210432 36056915

[37] Li, S. J. et al. Hierarchical micro/nanostructured silver hollow fiber boosts electroreduction of carbon dioxide. *Nat. Commun.* **13**, 3080 (2022). 35654817 10.1038/s41467-022-30733-6 PMC9163090

[38] Zhu, C. et al. Ampere-level CO_2_ reduction to multicarbon products over a copper gas penetration electrode. *Energy Environ. Sci.* **15**, 5391–5404 (2022). 10.1039/D2EE02121H

[39] Chen, A. et al. Gas penetrating hollow fiber Bi with contractive bond enables industry-level CO_2_ electroreduction. *Appl. Catal. B-Environ.* **333**, 122768 (2023). 10.1016/j.apcatb.2023.122768

[40] Sheng, X., Ge, W., Jiang, H. & Li, C. Engineering the Ni-N-C catalyst microenvironment enabling CO_2_ electroreduction with nearly 100% CO selectivity in acid. *Adv. Mater.* **34**, 2201295 (2022). 10.1002/adma.202201295 35901104

[41] Jiang, Z. et al. Molecular catalyst with near 100% selectivity for CO_2_ reduction in acidic electrolytes. *Adv. Energy Mater.* **13**, 2203603 (2022). 10.1002/aenm.202203603

[42] O’Brien, C. P. et al. Single pass CO_2_ conversion exceeding 85% in the electrosynthesis of multicarbon products via local CO_2_ regeneration. *ACS Energy Lett.* **6**, 2952–2959 (2021). 10.1021/acsenergylett.1c01122

[43] Rosen, J. et al. Mechanistic insights into the electrochemical reduction of CO_2_ to CO on nanostructured Ag surfaces. *ACS Catal.* **5**, 4293–4299 (2015). 10.1021/acscatal.5b00840

[44] Dunwell, M., Luc, W., Yan, Y. S., Jiao, F. & Xu, B. J. Understanding surface-mediated electrochemical reactions: CO_2_ reduction and beyond. *ACS Catal.* **8**, 8121–8129 (2018). 10.1021/acscatal.8b02181

[45] Ma, Z. et al. CO_2_ electroreduction to multicarbon products in strongly acidic electrolyte via synergistically modulating the local microenvironment. *Nat. Commun.* **13**, 7596 (2022). 36494381 10.1038/s41467-022-35415-x PMC9734127

[46] Liu, M. et al. Enhanced electrocatalytic CO_2_ reduction via field-induced reagent concentration. *Nature* **537**, 382–386 (2016). 27487220 10.1038/nature19060

[47] Hori, Y., Wakebe, H., Tsukamoto, T. & Koga, O. Electrocatalytic process of CO selectivity in electrochemical reduction of CO_2_ at metal electrodes in aqueous media. *Electrochimica Acta* **39**, 1833–1839 (1994). 10.1016/0013-4686(94)85172-7

[48] Gu, J., Hsu, C. S., Bai, L. C., Chen, H. M. & Hu, X. L. Atomically dispersed Fe^3+^ sites catalyze efficient CO_2_ electroreduction to CO. *Science* **364**, 1091–1094 (2019). 31197014 10.1126/science.aaw7515

[49] Fan, M. et al. Cationic-group-functionalized electrocatalysts enable stable acidic CO_2_ electrolysis. *Nat. Catal.* **6**, 763–772 (2023). 10.1038/s41929-023-01003-5

[50] Wei, D. X. et al. Decrypting the controlled product selectivity over Ag-Cu bimetallic surface alloys for electrochemical CO_2_ reduction. *Angew. Chem. Int. Ed.* **62**, e202217369 (2023). 10.1002/anie.202217369 36916416

[51] Kresse, G. & Joubert, D. From ultrasoft pseudopotentials to the projector augmented-wave method. *Phys. Rev. B* **59**, 1758–1775 (1999). 10.1103/PhysRevB.59.1758

[52] Perdew, J. P., Burke, K. & Ernzerhof, M. Generalized gradient approximation made simple. *Phys. Rev. Lett.* **77**, 3865–3868 (1996). 10062328 10.1103/PhysRevLett.77.3865

[53] Monkhorst, H. J. & Pack, J. D. Special points for brillouin-zone integrations. *Phys. Rev. B* **13**, 5188–5192 (1976). 10.1103/PhysRevB.13.5188

