# Supplementary material for: Ampere-level CO2 electroreduction with single-pass conversion exceeding 85% in acid over silver penetration electrodes
Source: Nat Commun. 2024 Jul 19;15:6101. doi: 10.1038/s41467-024-50521-8 (PMC11271590; doi:10.1038/s41467-024-50521-8)
Supplement: Supplementary file 1 — Supplementary Information [file 41467_2024_50521_MOESM1_ESM.pdf]

Supplementary Information for

Ampere-level CO_2 electroreduction with single-pass conversion exceeding 85% in acid over silver penetration electrodes

Shoujie Li[1,2#], Xiao Dong[1,2#], Gangfeng Wu[1,2#], Yanfang Song[1,2], Jianing Mao[1,3], Aohui Chen[1,2,4], Chang Zhu[1,2], Guihua Li[1,2], Yiheng Wei[1,2], Xiaohu Liu[1,2,4], Jiangjiang Wang[1,2], Wei Chen[1,2*] and Wei Wei[1,2,4*]

[1]Low-Carbon Conversion Science and Engineering Center, Shanghai Advanced Research Institute, Chinese Academy of Sciences, Shanghai 201210, China
[2]State Key Laboratory of Low Carbon Catalysis and Carbon Dioxide Utilization, Shanghai Advanced Research Institute, Chinese Academy of Sciences, Shanghai 201210, China
[3]Shanghai Institute of Applied Physics, Chinese Academy of Sciences, Shanghai 201204, China
[4]School of Physical Science and Technology, ShanghaiTech University, Shanghai 201203, China
[#]S.L., X.D. and G.W. contributed equally to this paper
*e-mail: chenw@sari.ac.cn; weiwei@sari.ac.cn

Supplementary Figures

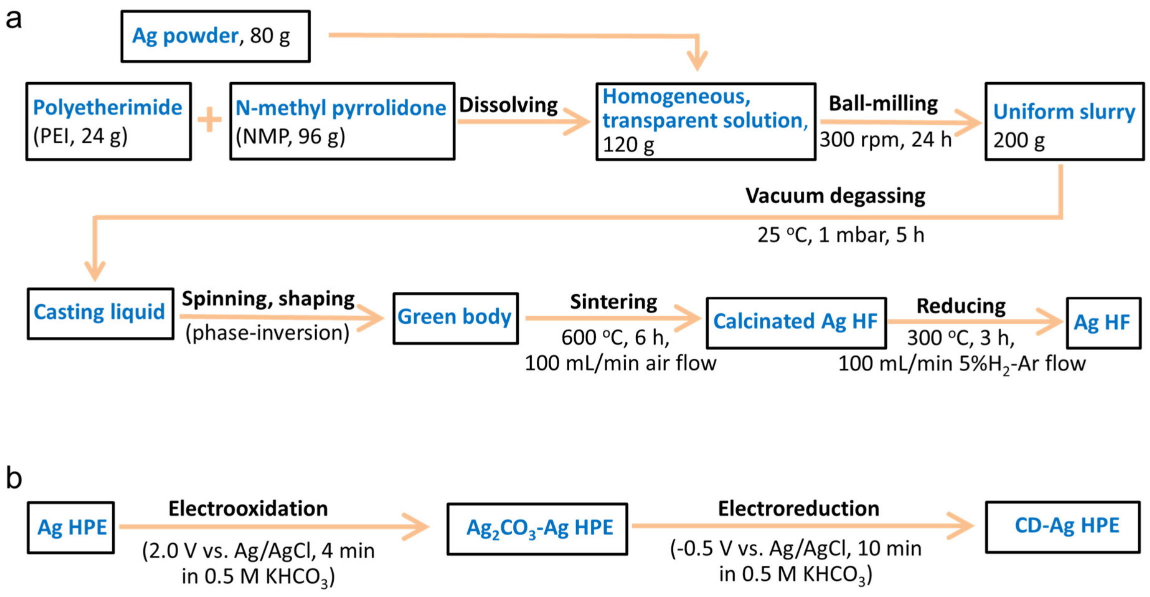

Supplementary Figure 1. Diagrams of the detailed fabrication procedures of **a**, Ag HPE and **b**, CD-Ag HPE.

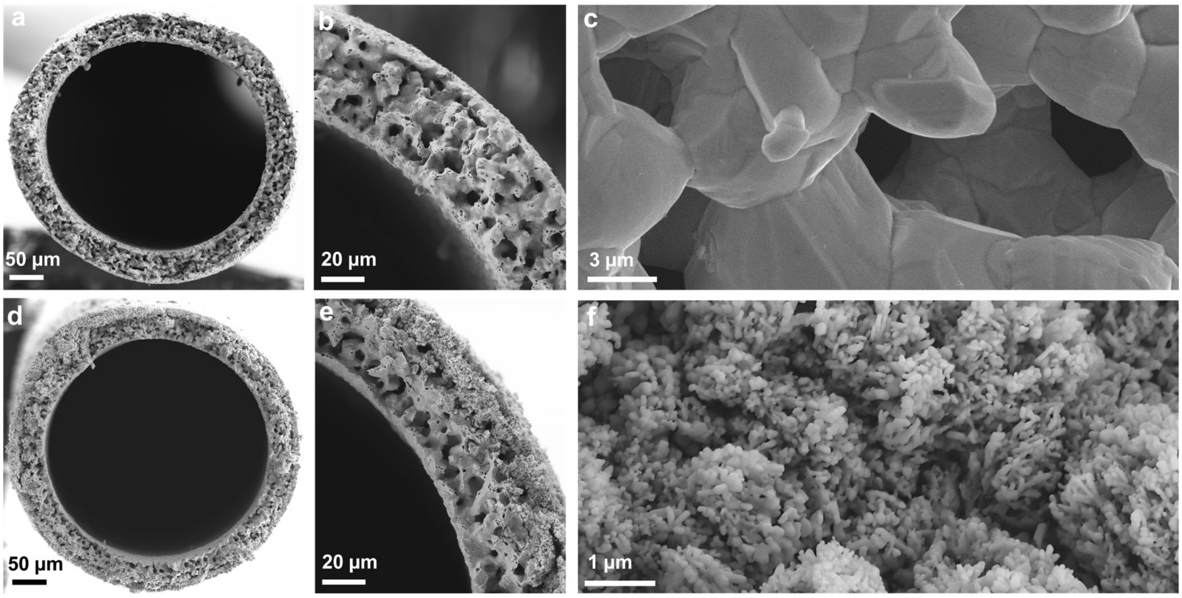

Supplementary Figure 2. Cross-section SEM images at **a, d,** low and **b, e,** high magnifications as well as **c, f,** surface mophology of **a-c** Ag HPE, **d-f** CD-Ag HPE. The SEM images of Supplementary Figs. 2**c, d, f,** same as Fig. 2**b, a, c** in the main text.

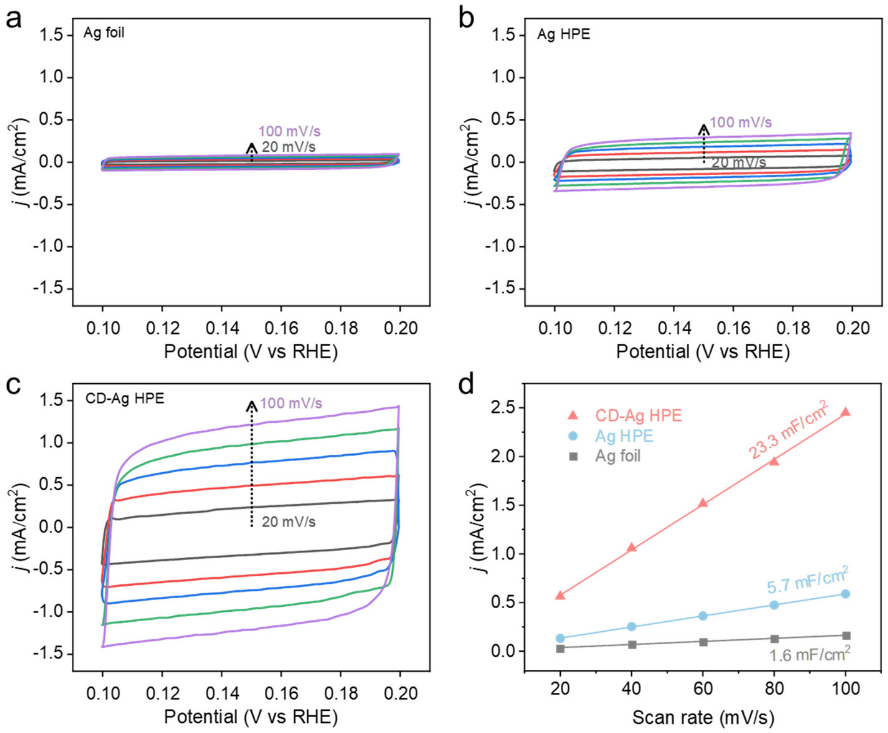

Supplementary Figure 3. Cyclic voltammetry curves of **a**, Ag foil, **b,** Ag HPE, **c,** CD-Ag HPE, **d,** Plot of Δj (the difference of cathodic and anodic current densities, j_c–j_a) against the scan rates from cyclic voltammetry curves.

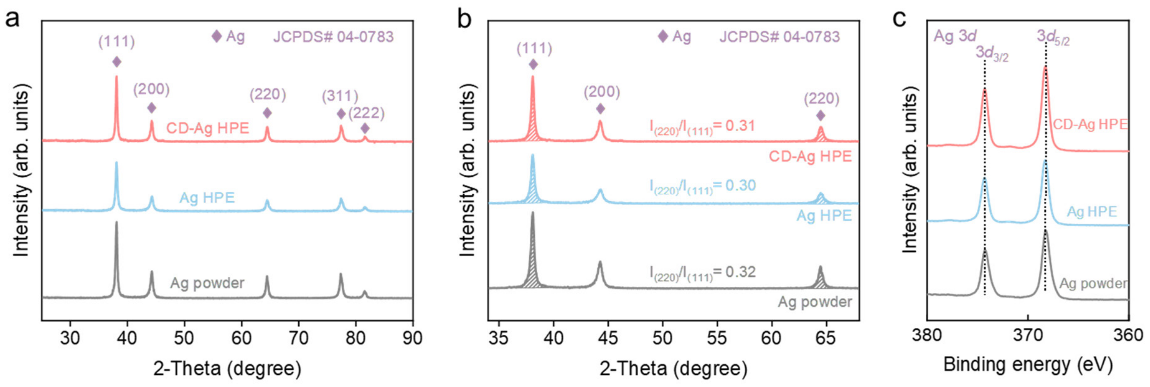

Supplementary Figure 4. a, b, The XRD patterns and **c,** XPS spectra of the Ag 3*d* level of Ag powder, Ag HPE and CD-Ag HPE. The $I_{(111)}$ and $I_{(220)}$ in Supplementary Fig. 4**b** represent the integral values of Ag (111) and Ag (220) peaks, respectively. The plots of Ag HPE and CD-Ag HPE in Supplementary Fig. 4**a, c**, same as Fig. 2**d, e,** in the main text.

In order to characterize whether CD-Ag HPE had a higher proportion of Ag (110) orientation than Ag HPE, we integrated their XRD peaks of Ag (111) and Ag (220) (to be note that the fact that Ag (220) is parallel to Ag (110) in a face-centered cubic, so it's roughly equal to Ag (110)). The result showed that there was no significant difference in the ratio of $I_{(220)}/I_{(111)}$ between CD-Ag HPE and Ag HPE (Supplementary Fig. 4**b**), indicating there was no obvious crystal plane orientation.

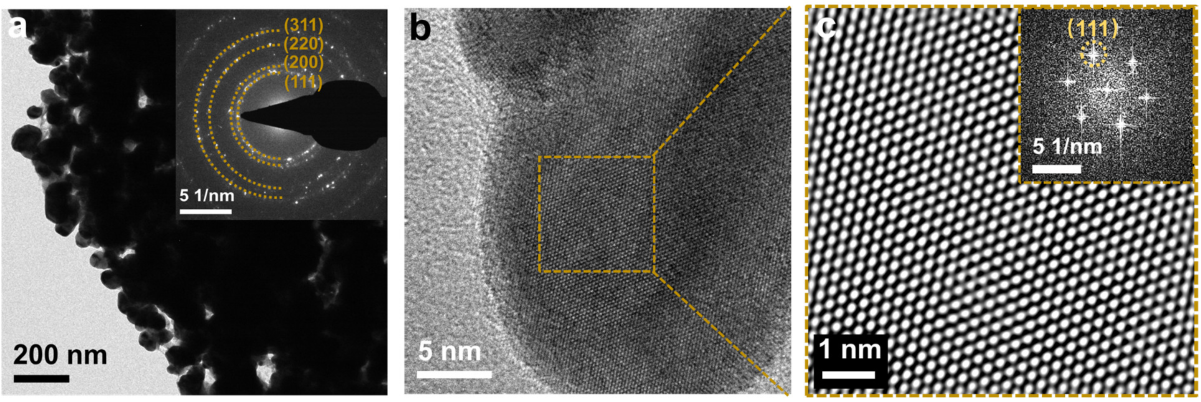

Supplementary Figure 5. a, TEM image and corresponding SAED pattern (insert of **a**), (**b, c,**) high-magnification TEM images and corresponding FFT image of CD-Ag HPE (insert of **c**). The images of CD-Ag HPE in Supplementary Fig 5**a**, same as Fig. 2**f** in the main text.

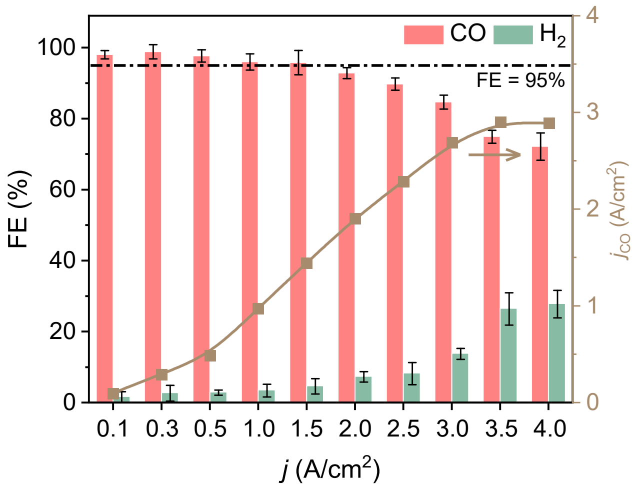

Supplementary Figure 6. The neutral CO_2RR performance of CD-Ag HPE in CO_2-saturated 3.0 M KCl + 0.01 M $KHCO_3$ (pH =6.6) catholyte at different current density (0.1-4 A/cm^2). The error bars represent one standard deviation based on five independent tests.

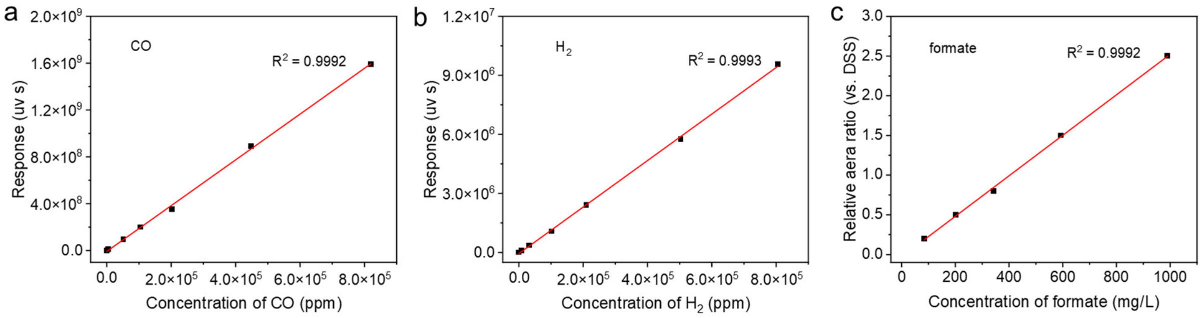

Supplementary Figure 7. Standard correlation curves and the corresponding linear correlation coefficients of **a,** CO, **b,** H_2 detected by GC and **c,** formate detected by ^{1}H NMR.

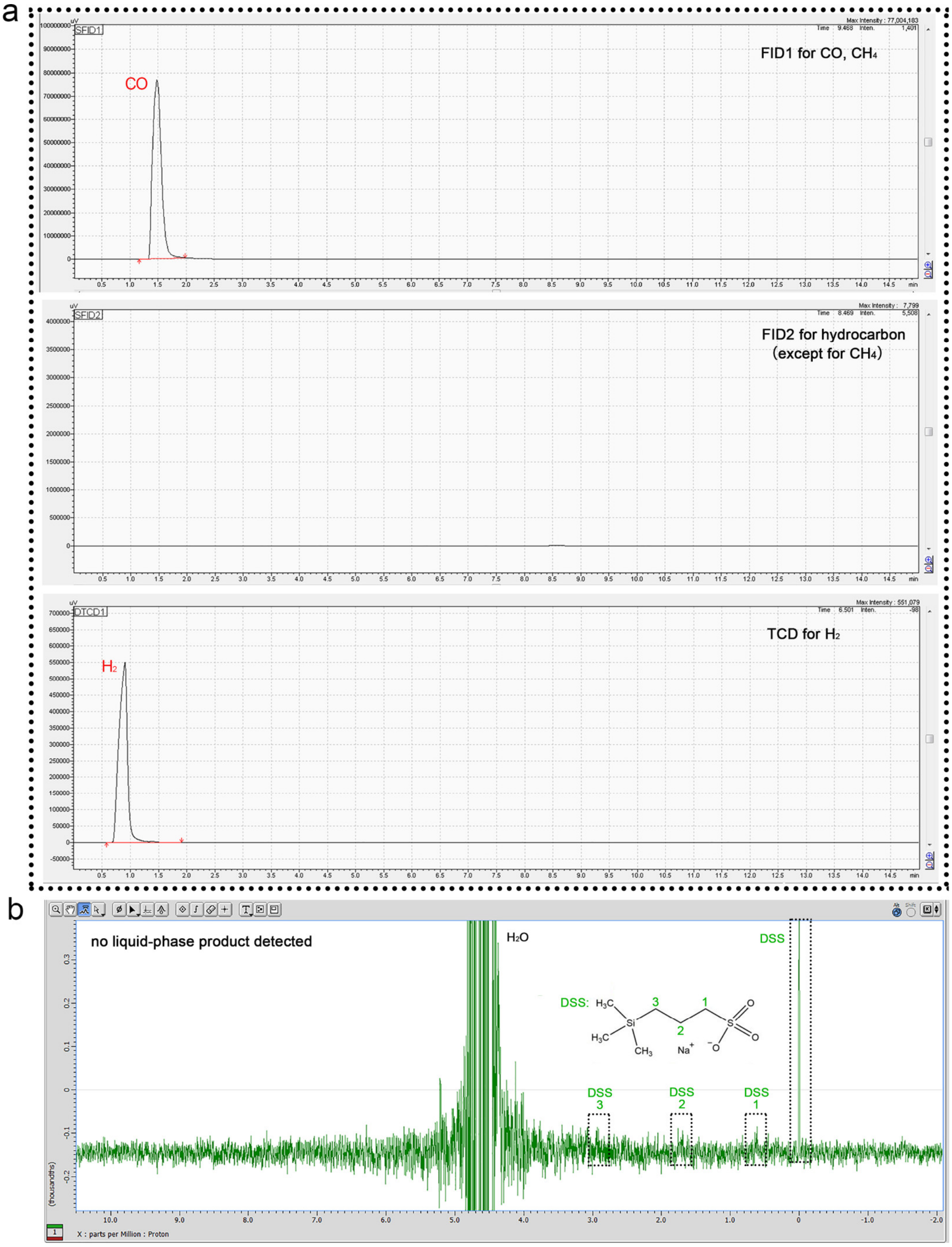

Supplementary Figure 8. a, GC curves and **b,** ^{1}H NMR spectrum of the catholyte after 1 h CO_2RR over CD-Ag HPE at 2 A/cm^2 (operated in a CO_2-saturated 0.05 M H_2SO_4 + 3 M KCl catholyte and 0.05 M H_2SO_4 + 0.5 M K_2SO_4 anolyte). The online GC results showing only CO and H_2 as gas-phase products, and ^{1}H NMR spectrum showing no liquid-phase product formed.

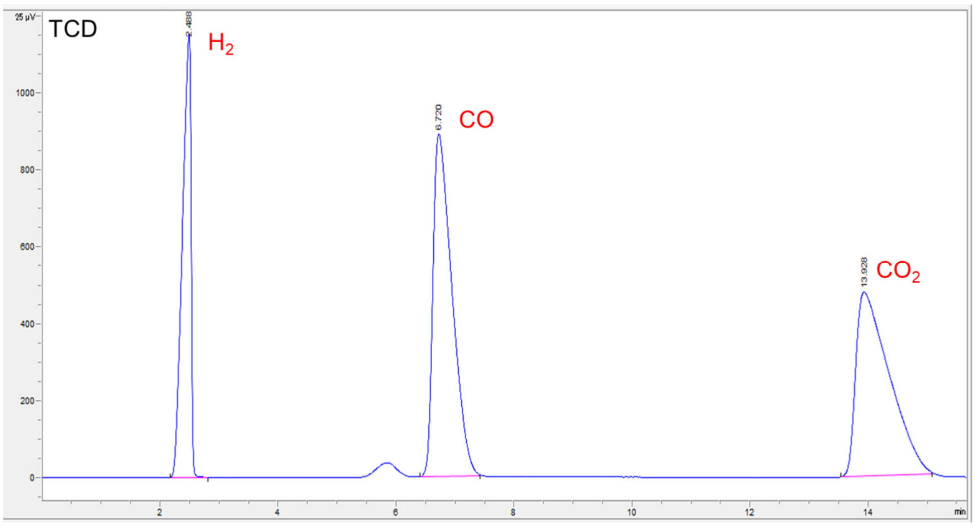

Supplementary Figure 9. The online GC curve from TCD over CD-Ag HPE at 2 A/cm^2 (operated in a CO_2-saturated 0.05 M H_2SO_4 + 3 M KCl catholyte and 0.05 M H_2SO_4 + 0.5 M K_2SO_4 anolyte). The concentrations of H_2, CO and CO_2 could be quantified in TCD Channel.

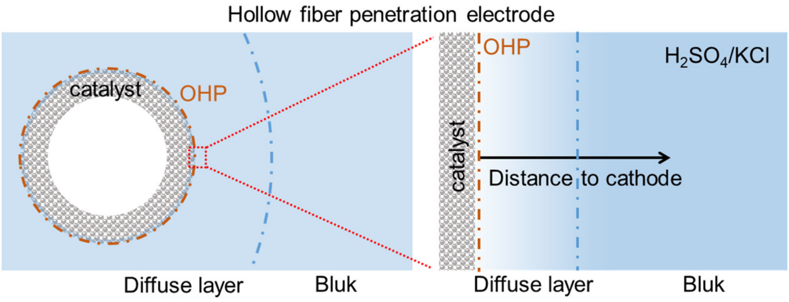

Supplementary Figure 10. Schematic of a one-dimensional (1D) COMSOL models of working electrode of hollow fiber penetration electrode. Outer Helmholtz Plane (OHP). A reaction-diffusion model was used to simulate the local pH and CO_2 concentration using COMSOL Multiphysics software in a typical 50 μm diffusion layer. One end of the one-dimensional simulation area is set as the working electrode surface, and the other side is set as the bulk concentration to describe the bulk electrolyte.

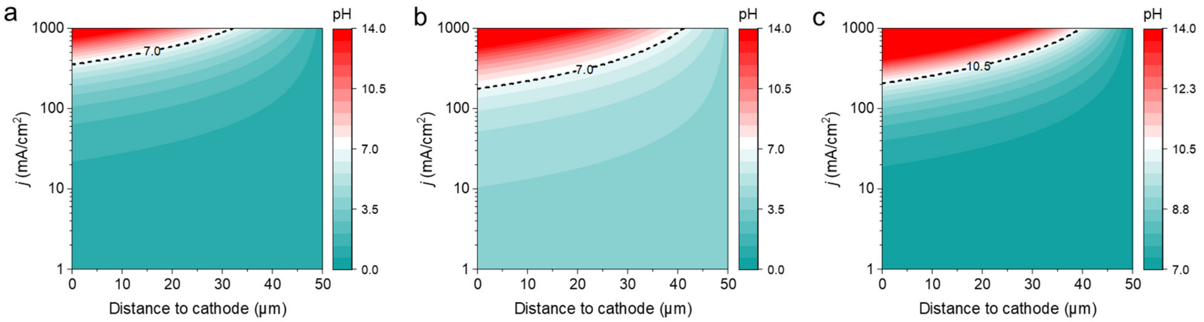

Supplementary Figure 11. Modeling of pH at different distances to cathode and current density in H_2SO_4 with 3 M KCl at pH **a,** 1, **b,** 4, **c,** 7.

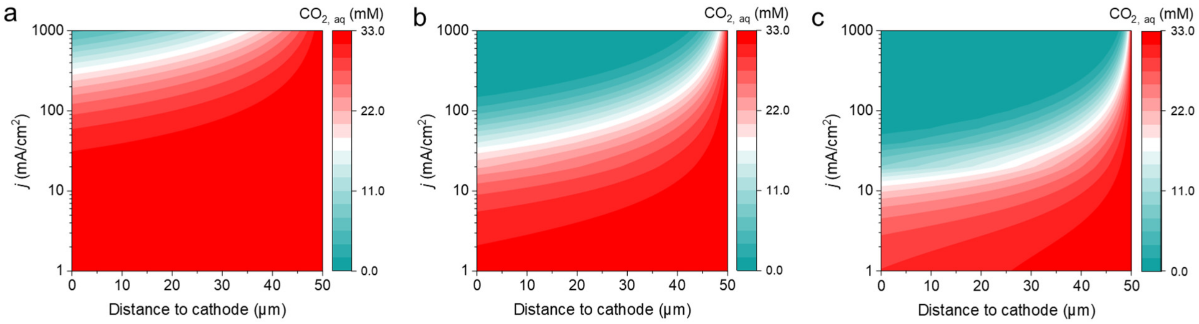

Supplementary Figure 12. Modeling of concentration profile of CO_2 at different distances to cathode and current density in H_2SO_4 with 3 M KCl at pH **a,** 1, **b,** 4, **c,** 7.

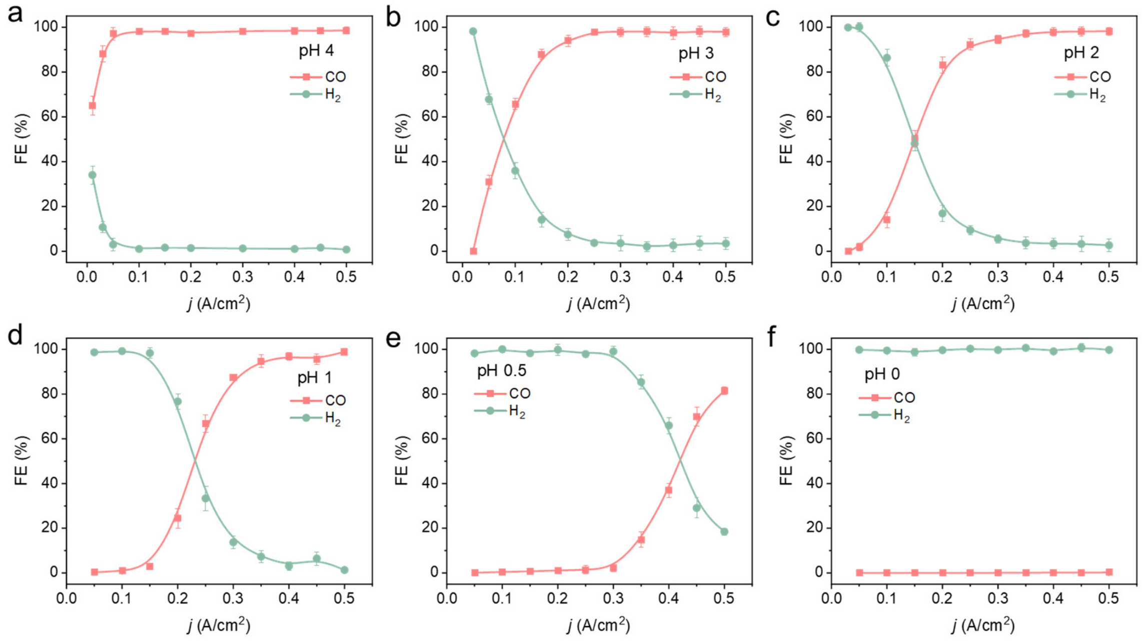

Supplementary Figure 13. The CO_2RR performance of CD-Ag HPE in 3 M KCl with **a,** pH 4, **b,** pH 3, **c,** pH 2, **d,** pH 1, **e,** pH 0.5 and **f,** pH 0 at low current density (0-0.5 A/cm^2). The error bars represent one standard deviation based on five independent tests.

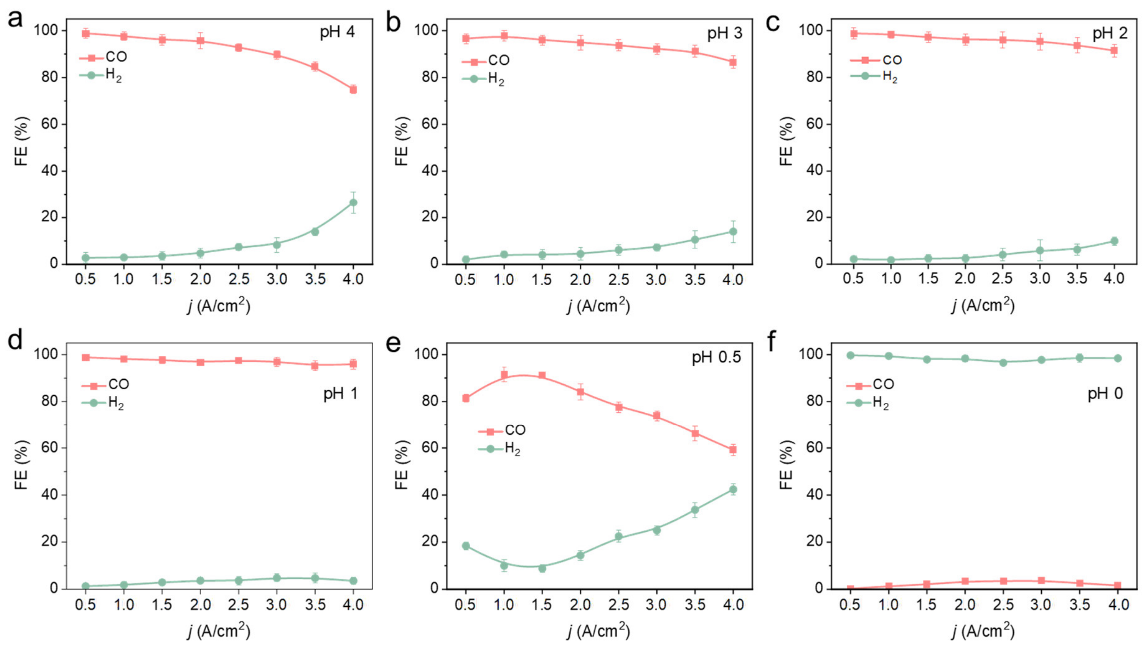

Supplementary Figure 14. The CO_2RR performance of CD-Ag HPE in 3 M KCl with **a**, pH 4, **b**, pH 3, **c**, pH 2, **d**, pH 1, **e**, pH 0.5 and **f**, pH 0 at high current density (0.5-4 A/cm^2). The error bars represent one standard deviation based on five independent tests.

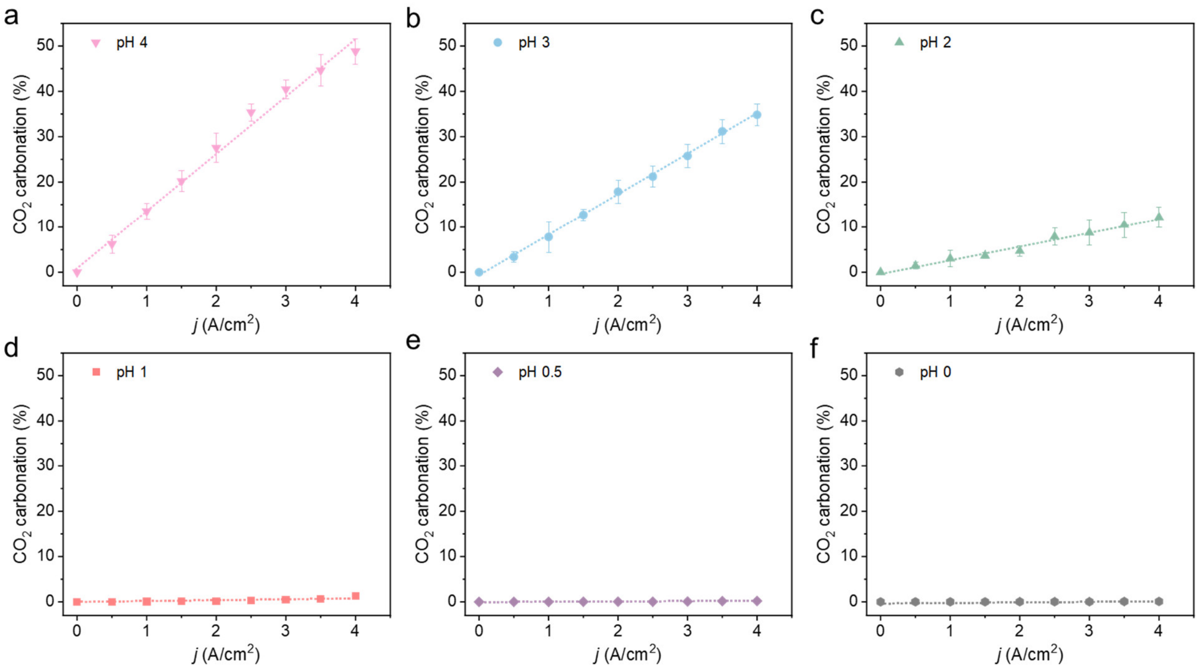

Supplementary Figure 15. CO_2 carbonation percentages over CD-Ag HPE as a function of applied current density measured in 3 M KCl with **a**, pH 4, **b**, pH 3, **c**, pH 2, **d**, pH 1, **e**, pH 0.5 and **f**, pH 0. The error bars represent one standard deviation based on five independent tests.

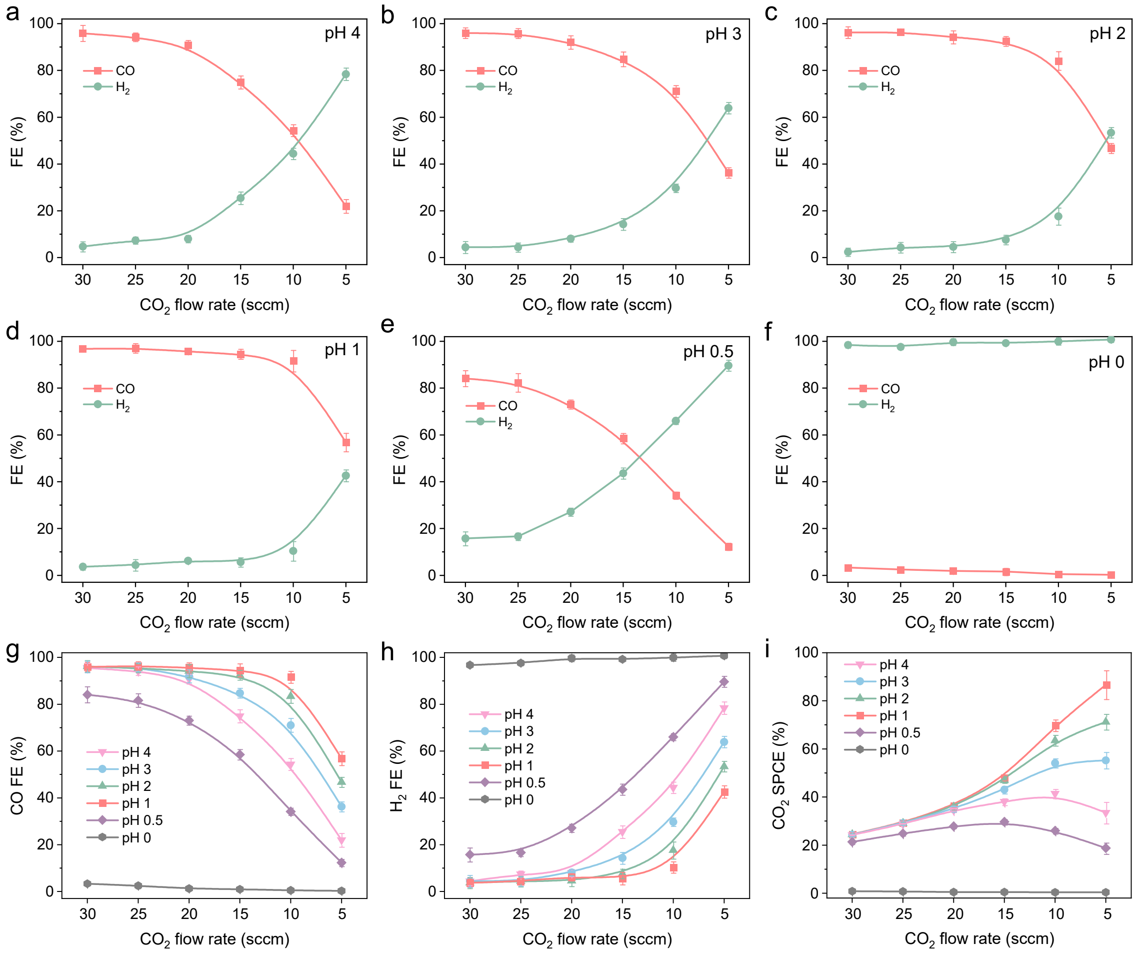

Supplementary Figure 16. The CO_2RR performance of CD-Ag HPE as a function of input CO_2 flow rate in 3 M KCl with **a**, pH 4, **b**, pH 3, **c**, pH 2, **d**, pH 1, **e**, pH 0.5 and **f**, pH 0 at a constant current density of 2 A/cm^2. **g**, CO, **h**, H_2 FE and **i**, CO_2 SPCE over CD-Ag HPE as a function of input CO_2 flow rate in 3 M KCl + H_2SO_4 catholytes with different pH values at a constant current density of 2 A/cm^2. The plots of CD-Ag HPE in Supplementary Fig. 16**g, i**, same as Fig. 4**c, e,** in the main text. The error bars represent one standard deviation based on five independent tests.

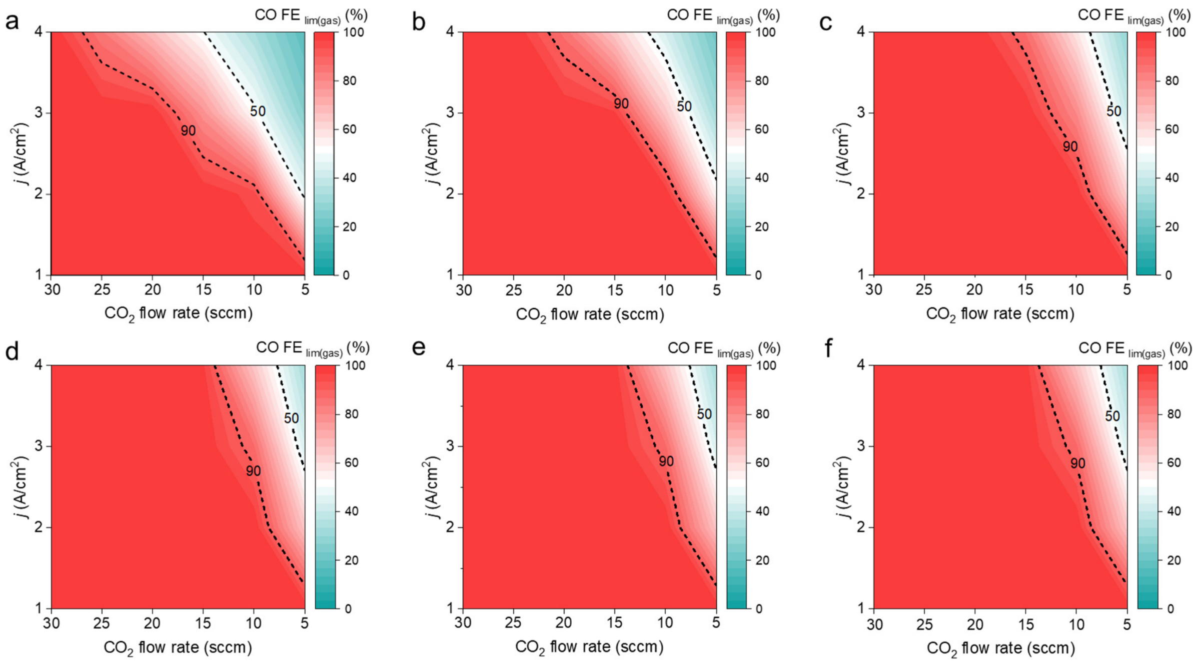

Supplementary Figure 17. The *j*–CO_2 flow rate-dependent mapping distribution of theoretical CO FE limit value at pH **a**, 4, **b**, 3, **c**, 2, **d**, 1, **e**, 0.5, **f**, 0.

To further investigate the impact of the concentrations of H^+ and local availability of CO_2 on CO_2RR performance at high *j* of CD-Ag HPE, the *j*–CO_2 flow rate-dependent mapping distribution of theoretical CO FE limit based on CO_2 carbonation in different pH electrolyte were plotted. As shown in the Supplementary Fig. 17, at a high flow rate of 30 mL/min, the theoretical CO FE limit values of all pH electrolytes (0-4) could basically reach or approach 100% even at *j* as high as 4 A/cm^2, which is consistent with the experimental results (Fig. 3**e, f**). However, compared with the theoretical CO FE limit values of pH 1, 0.5 and 0 with basically no CO_2 carbonation (Supplementary Fig. 17**d-f**), the theoretical CO FE limit values of pH 4, 3 (Supplementary Fig. 17**a, b**) rapidly decreases with the decreasing of input CO_2 flow rates and the increasing of *j*. In particular, at pH 4, when the *j* >2 A/cm^2, as long as the CO_2 flow rates <25 sccm, the theoretical CO FE limit values were <90% (Supplementary Fig. 17**a**). The theoretical CO FE limit values

were even less than 50% at the j higher than 3 A/cm^2, where the HER dominated. In contrast, at a pH of 1 (Supplementary Fig. 17**d**), higher theoretical CO FE limit values could be achieved at lower input CO_2 flow rates and higher j, indicating higher theoretical limit j_{CO} as well as a higher CO_2 SPCE could be achieved only in a strong acidic electrolyte.

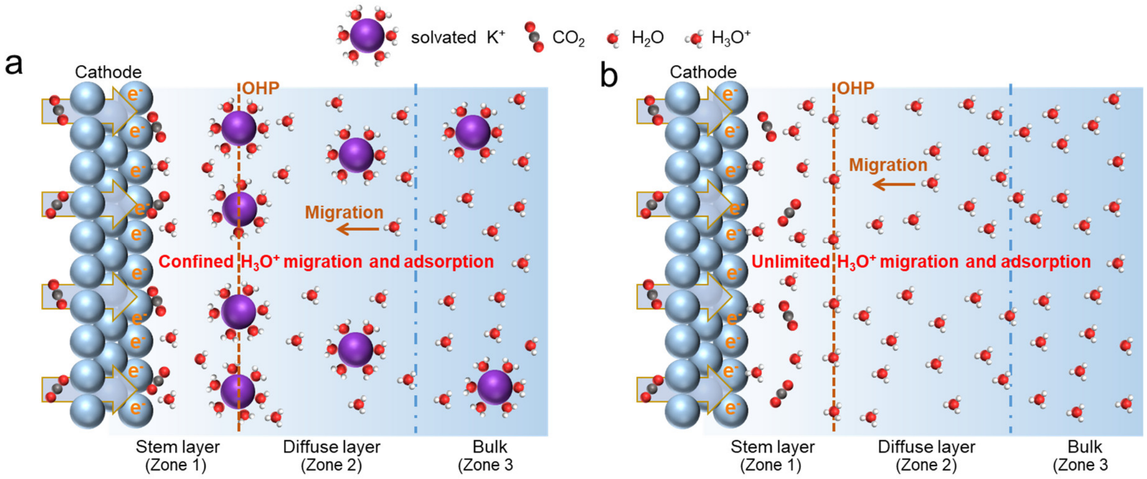

Supplementary Figure 18. Schematic diagram of electric double layer near cathode in **a**, H_2SO_4 + KCl and **b**, H_2SO_4 electrolytes during CO_2RR. Outer Helmholtz Plane (OHP).

In K^+-containing acidic electrolyte, due to the competitive adsorption of hydrated K^+ against H^+ at OHP, a chemically inert hydrated K^+ layer formed at OHP and shielded the electric field from the cathode in a long potential window[1,2]. Thus, migration of H^+ was dramatically suppressed, which lowers the concentration of H^+ in the OHP and thus suppresses HER. Meanwhile hydrated K^+ strengthen the field in the Stern layer and stabilize key intermediates in CO_2 reduction. The effect of this shielding electric field is enhanced with the increase of K^+ concentration.

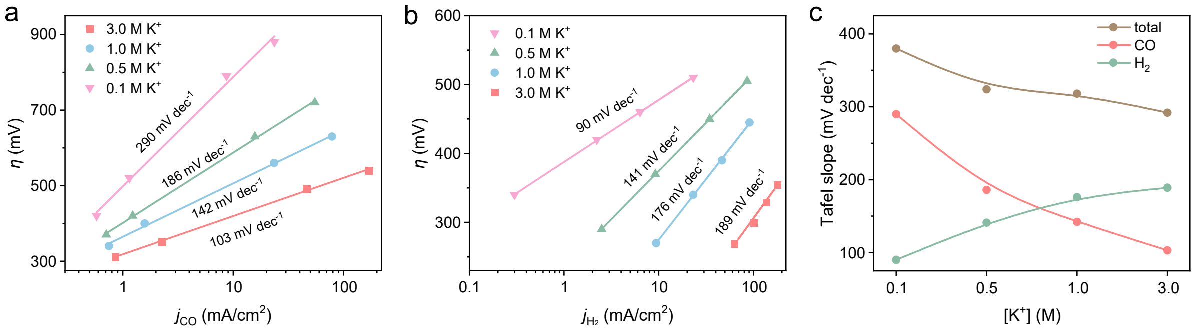

Supplementary Figure 19. Tafel slopes of **a**, CO, **b**, H_2 and **c**, their values variation with different K^+ concentrations at pH 1. The plots of CD-Ag HPE in Supplementary Figure 19a, same as Fig. 5b, in the main text.

In general, the initial step in the overall two-electron reduction of CO_2 to CO on the silver surface was a one-electron transfer step forming adsorbed $*COO^-$ (Step 1). Then, a chemical step involving the protonation of $*COO^-$ to form a *COOH intermediate (Step 2). Subsequently, an electrochemical path coupled to a chemical reaction involving the proton-electron transfer and instantaneous dehydration to form an adsorbed *CO intermediate (Step 3). Finally, the desorption of *CO from the silver surface to obtain the CO product (Step 4). According to the previous reports[3,4], if Step 1 was the RDS for the electrode, the Tafel slope will be generally about 118 $mV \cdot dec^{-1}$, and the Tafel slope will be generally close to 59 $mV \cdot dec^{-1}$, if Step 2 was the RDS. Then, if Step 3 was the RDS, the Tafel slope will be generally less than 40 $mV \cdot dec^{-1}$, and the Tafel slope will be ∞ (infinity), if Step 4 was the RDS[3,4]. In general, the lower Tafel slope value means that the electrode reelectrolysis process has faster electron transfer capacity and better electrocatalytic kinetics. In this work, with the increase of K^+ concentration, we observed that the Tafel slope values of CO gradually decreased (Fig. 5**b**), indicating that the capacity of electron transfer upon CO_2 adsorption was enhanced, and the kinetics of CO_2RR to CO was improved. At the same time, it can be seen that the H_2 Tafel slope value increased with increasing of K^+ concentrations,

indicating that the presence of K^+ would suppress the HER kinetics. In addition, the total of Tafel slope values of CO and H_2 gradually decreased with increasing of K^+ concentrations (Supplementary Fig. 19**c**), which was consistent with the results in Fig. 5**c**, where a faster electron transfer was verified by the lowest interfacial charge transfer resistance (R_{ct}) of CD-Ag HPE in 3 M K^+ (0.9 Ω cm^2, Fig. 5**c** and Supplementary Table 8). These observations indicated that the presence of K^+ in the acidic electrolyte not only suppressed the HER, but also stimulated CO_2 activation and conversion.

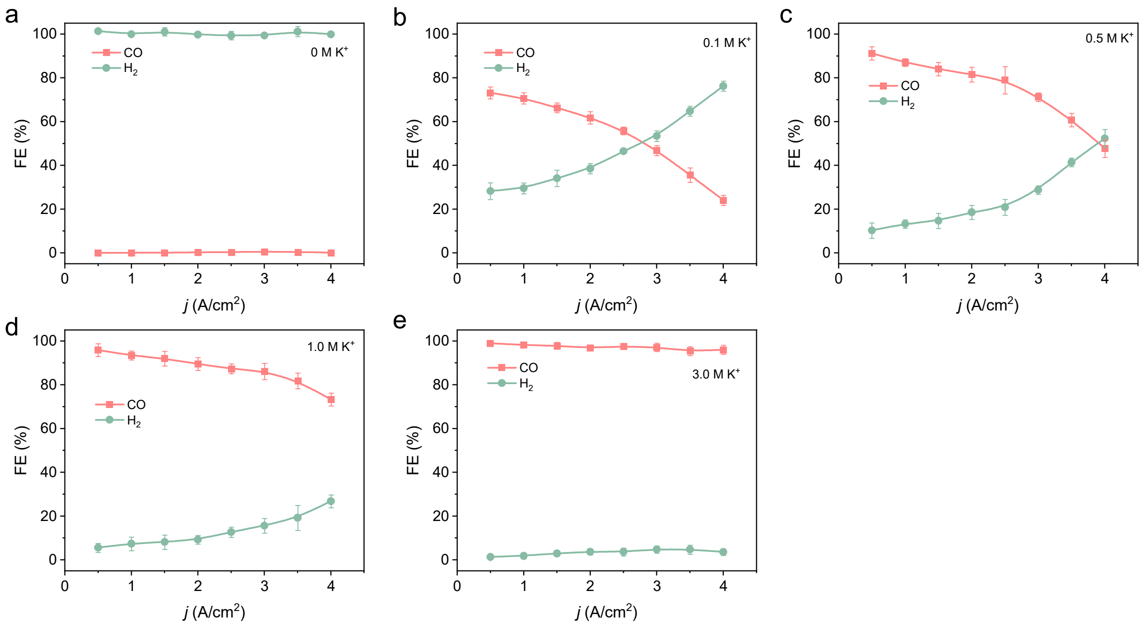

Supplementary Figure 20. The CO_2RR performance of CD-Ag HPE as a function of applied current density measured in H_2SO_4 catholytes with **a**, 0 M, **b**, 0.1 M, **c**, 0.5 M, **d**, 1.0 M, **e**, 3.0 M K^+ concentrations at pH 1 (input CO_2 flow rate: 30 sccm). The error bars represent one standard deviation based on five independent tests.

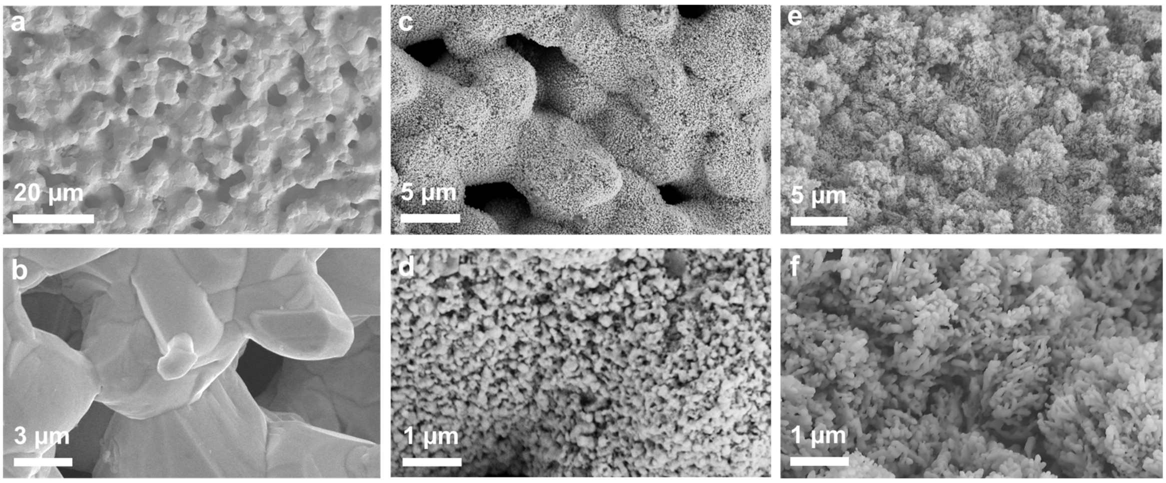

Supplementary Figure 21. The surface SEM images of **a**, **b**, Ag HPE, **c, d,** OD-Ag HPE and **e, f,** CD-Ag HPE. The SEM images of Supplementary Fig. 21**b, f**, same as Fig. 2**b, c** in the main text.

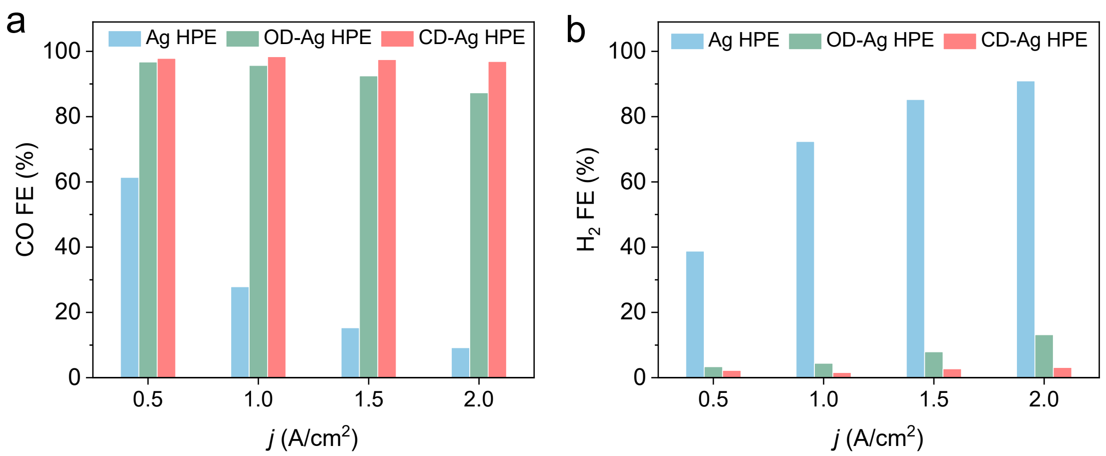

Supplementary Figure 22. The **a**, CO and **b,** H_2 faradaic efficiencies of Ag HPE, OD-Ag HPE and CD-Ag HPE in CO_2-saturated 3 M KCl + 0.05 M H_2SO_4 catholytes at different current densities (pH 1, input CO_2 flow rate: 30 sccm).

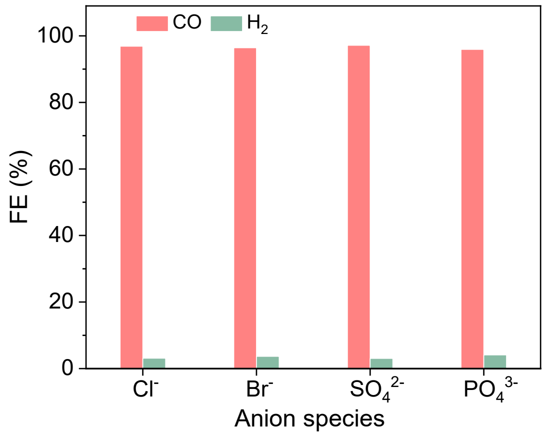

Supplementary Figure 23. Faradaic efficiency on the CD-Ag HPE under 2 A/cm^2 in electrolytes of similar pH with different anions species (pH 1).

As shown in Supplementary Fig. 23, there was no significant difference between the CO and H_2 faradaic efficiency of KCl, KBr, K_2SO_4 and K_3PO_4 under a constant current density of 2 A/cm^2, indicating the effect of anions on CO_2RR to CO reactivity was not significant in a strong acidic electrolyte. However, in a strong acidic electrolyte, when K^+ was absent, almost no CO_2RR occurs, and the performance of CO_2RR improves rapidly with the increasing of K^+ concentration, while HER was well suppressed (Fig. 5**d, e** and Supplementary Fig. 20). Previous work[4,5] and current experimental results indicate that the effect of Cl^- on promotion of CO_2RR to CO performance mainly occurs in the neutral electrolysis environment, while in a strong acidic electrolysis environment, the promotion effect of Cl^- on CO_2RR is not significant, and the significant improvement of CO_2RR to CO performance while inhibiting HER should be attributed to the presence of K^+ [6,7].

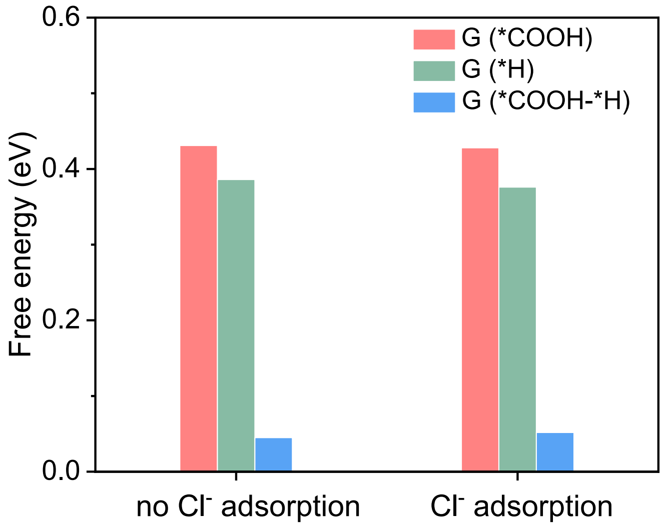

Supplementary Figure 24. The free energy for the formation of *COOH (G(*COOH)), *H (G(*H)), and their energy difference (G(*COOH)-G(*H)) on bare Ag (111) plane and Cl^- adsorption Ag (111) plane.

As shown in the Supplementary Fig. 24, under an acidic condition, the Gibbs free energy of *COOH and *H, the key intermediates from CO_2RR to CO and HER, respectively, basically did not change whether there was Cl^- adsorption or not. These DFT calculations are in agreement with the experimental results, indicating that in a strong acidic electrolysis environment, the improvement of Cl^- in electrolyte on CO_2RR is not significant.

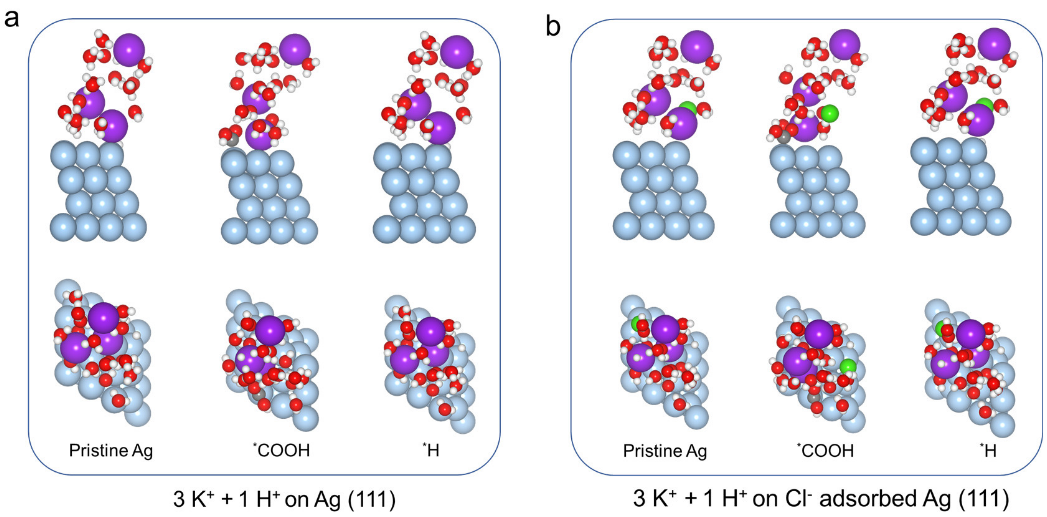

Supplementary Figure 25. The free energy for the formation of *COOH (G(*COOH)), *H (G(*H)), and their energy difference (G(*COOH)-G(*H)) on **a**, bare Ag (111) plane and **b**, Cl^- adsorption Ag (111) plane. The light blue, purple, green, red, gray and white balls represent Ag, K, Cl, O, C and H, respectively.

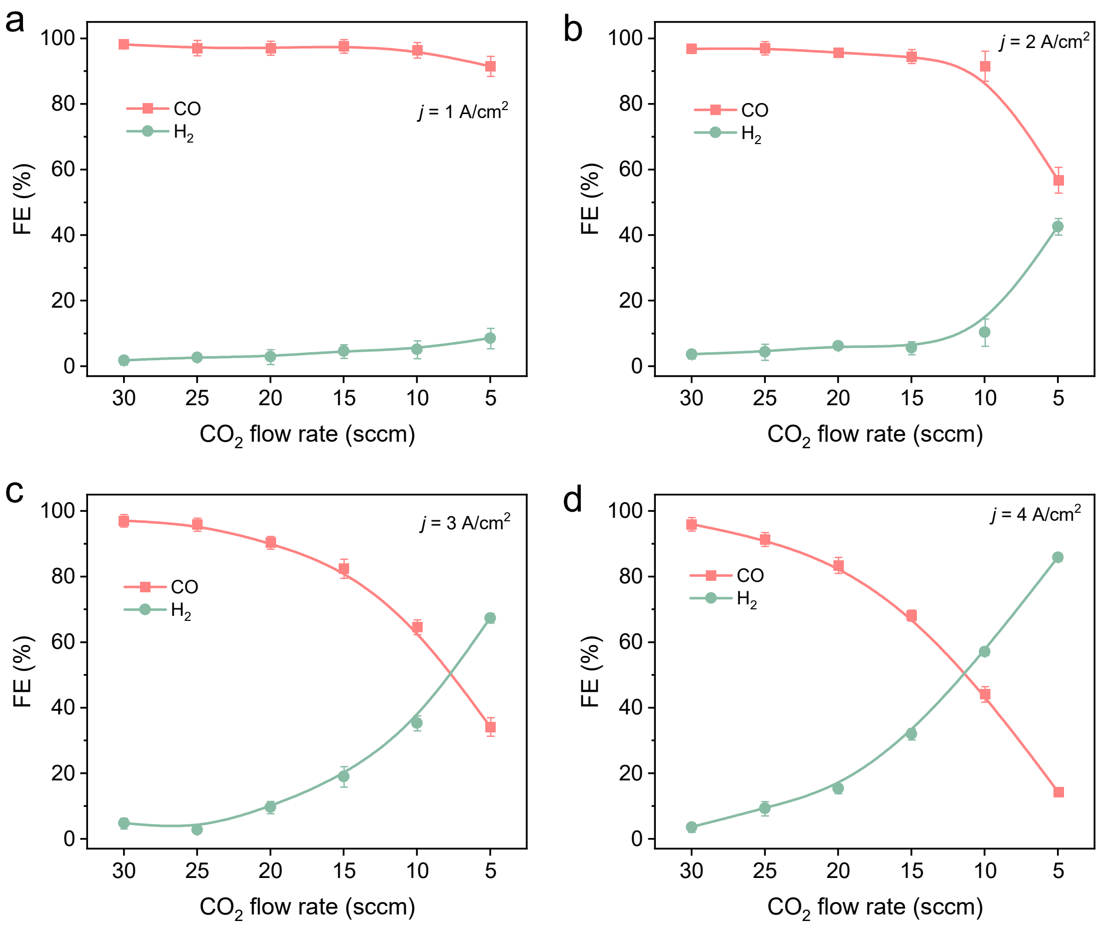

Supplementary Figure 26. The CO_2RR performance of CD-Ag HPE as a function of input CO_2 flow rate in CO_2-saturated 3 M KCl + 0.05 M H_2SO_4 catholytes (pH 1) at constant current density of **a**, 1 A/cm^2, **b**, 2 A/cm^2, **c**, 3 A/cm^2 and **d**, 4 A/cm^2. The error bars represent one standard deviation based on five independent tests.

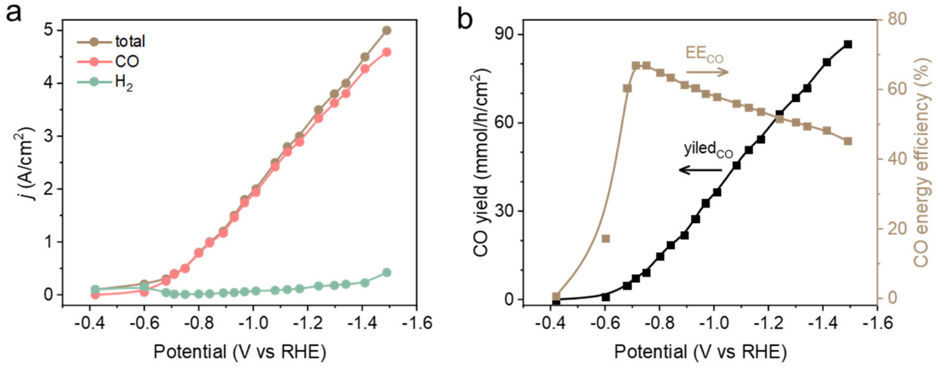

Supplementary Figure 27. a, Current densities of CO_2RR products and **b**, CO yiled and CO energy efficiency (details presented in Supplementary Table 2) on CD-Ag HPE as a function of potential measured in CO_2-saturated 3 M KCl + 0.05 M H_2SO_4 catholytes (pH 1, input CO_2 flow rate: 30 sccm).

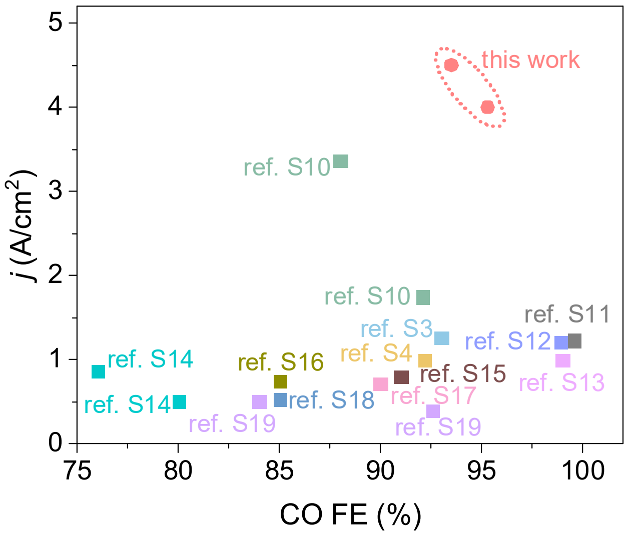

Supplementary Figure 28. Comparison of total current density and CO FE for the recently reported outstanding electrocatalysts for CO formation from CO_2 electroreduction.

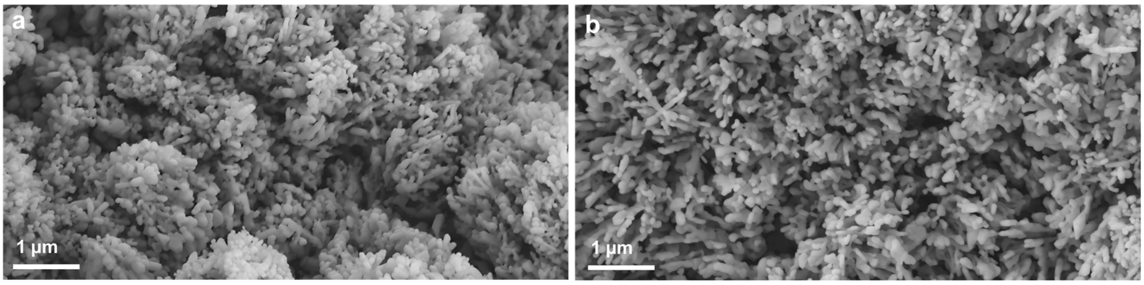

Supplementary Figure 29. The surface SEM images of **a**, CD-Ag HPE and **b**, postreaction CD-Ag HPE.

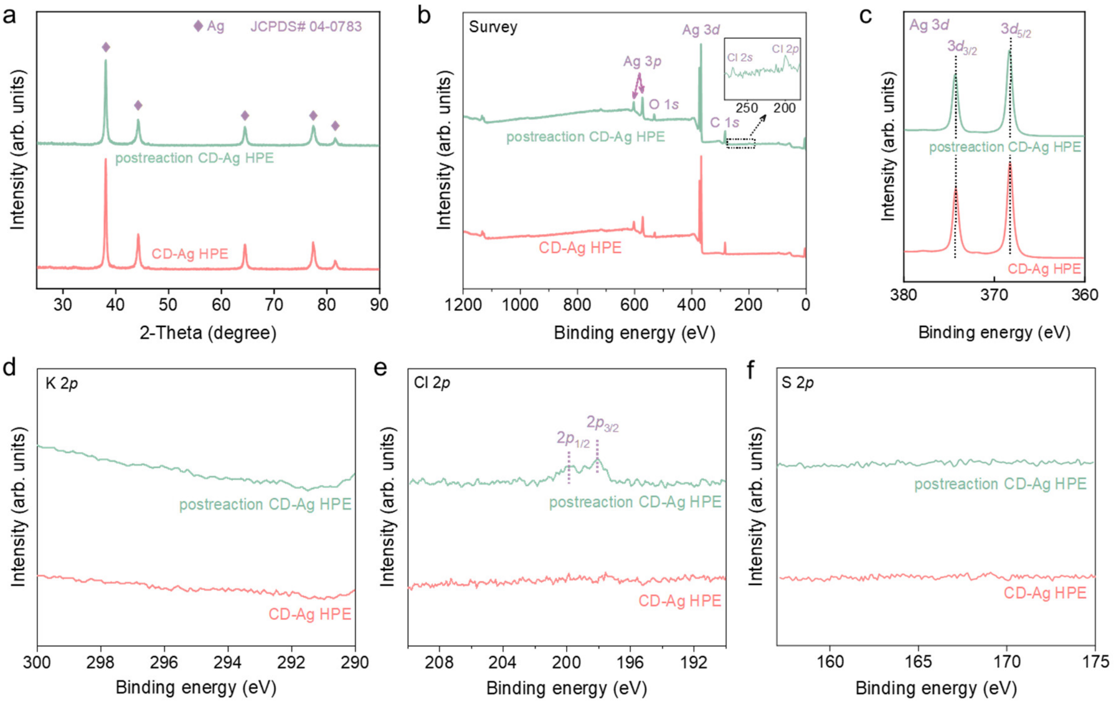

Supplementary Figure 30. a, The XRD patterns and **b-f**, XPS spectra of **b**, survey, **c**, Ag 3*d*, **d**, K 2*p*, **e**, Cl 2*p* and **f**, S 2*p* of CD-Ag HPE and postreaction CD-Ag HPE.

As shown in Supplementary Fig. 30**b**, compared to the pre-reaction data, no peaks of K and S were observed over the survey, except for very weak Cl 2*p* peaks that can be attributed to adsorption on the Ag surface. Furthermore, no any peaks of K and S were observed in the detailed fine spectra of K 2*p* (Supplementary Fig. 30**d**) and S 2*p* (Supplementary Fig. 30**f**), indicating that K and S elements did not affect the catalyst surface during the reaction. The appearance of weak Cl 2*p* peak in Supplementary Fig. 30**e** was consistent with previous reports[4,5], indicating the presence of Cl^- adsorption on the surface of the Ag electrode. Therefore, the results of XRD and XPS showed that only the weak Cl^- adsorption on surface did not change the surface and phase composition of CD-Ag HPE, and was still metallic Ag, indicating the stable metallic Ag^0 active component during CO_2 electroreduction.

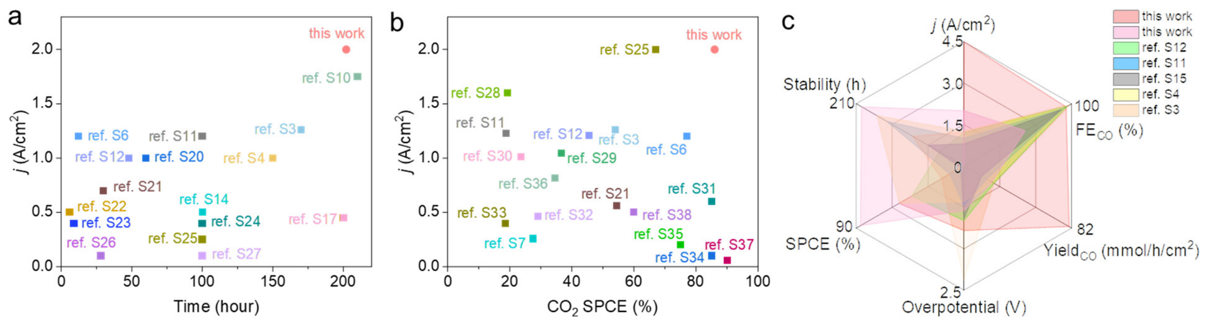

Supplementary Figure 31. Comparison of **a**, total current density and stability, **b**, total current density and CO2 SPCE for the recently reported outstanding CO2RR electrocatalysts.

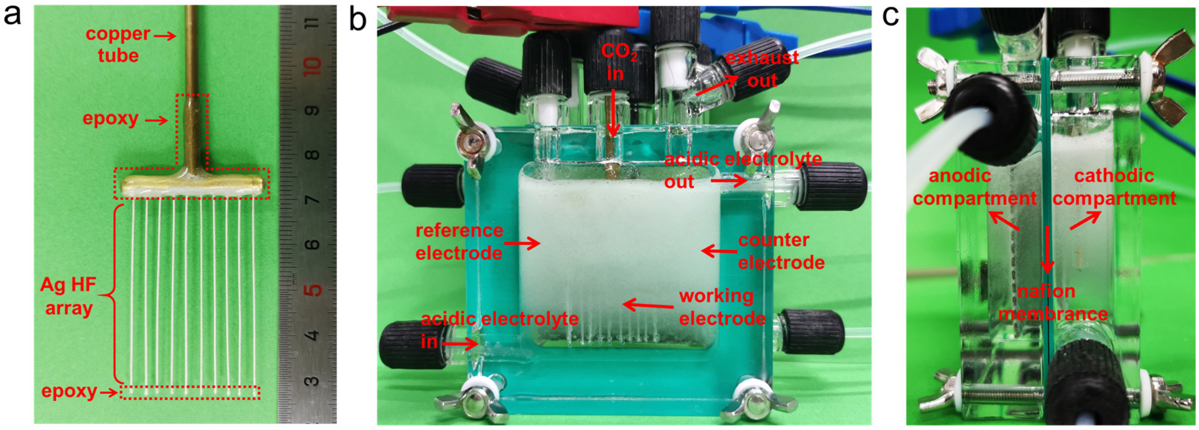

Supplementary Figure 32. Optical images of **a,** 10-tube CD-Ag HPE array electrode. The gas-tight two-compartment electrolysis cell **b**, from a side view, and **c,** from a cross-section view during CO_2 electroreduction. The arrows show the directions of the CO_2 flow, exhaust flow, and electrolyte solution flow.

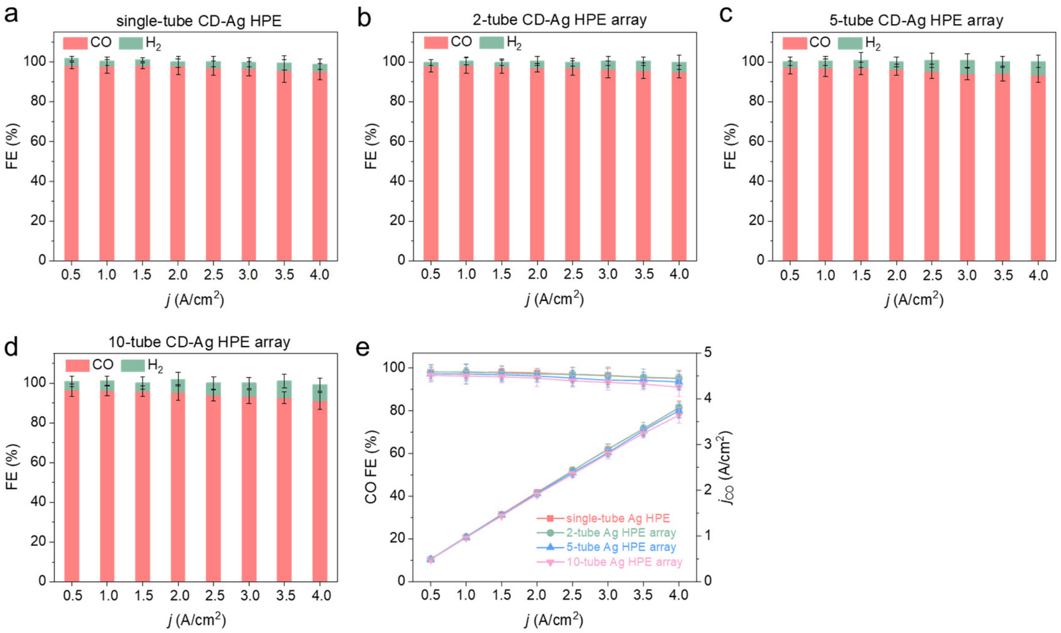

Supplementary Figure 33. The CO_2RR performance of **a**, single-tube CD-Ag HPE, **b**, 2-tube CD-Ag HPE array, **c**, 5-tube CD-Ag HPE array, and **d**, 10-tube CD-Ag HPE array at different current densities (0.5-4 A/cm^2) in a 2-electrode system with CO_2-saturated 3 M KCl + 0.05 M H_2SO_4 catholytes. **e,** CO FE and j_{CO} over single-tube CD-Ag HPE, 2-tube CD-Ag HPE array, 5-tube CD-Ag HPE array and 10-tube CD-Ag HPE array in 3 M KCl + H_2SO_4 catholytes at different current density. The error bars represent one standard deviation based on five independent tests.

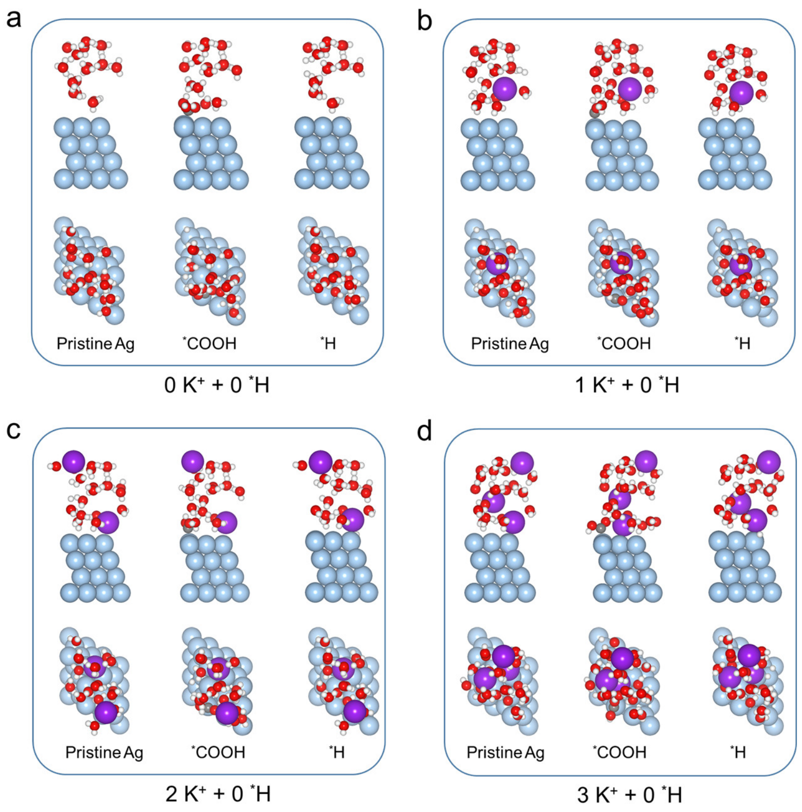

Supplementary Figure 34. The optimized periodic atomic structures under various K^+ concentrations (1/18 per H_2O molecule), **a**, 0 K^+ + 0 *H, **b**, 1 K^+ + 0 *H, **c**, 2 K^+ + 0 *H and **d**, 3 K^+ + 0 *H. The light blue, purple, red, gray and white balls represent Ag, K, O, C and H, respectively.

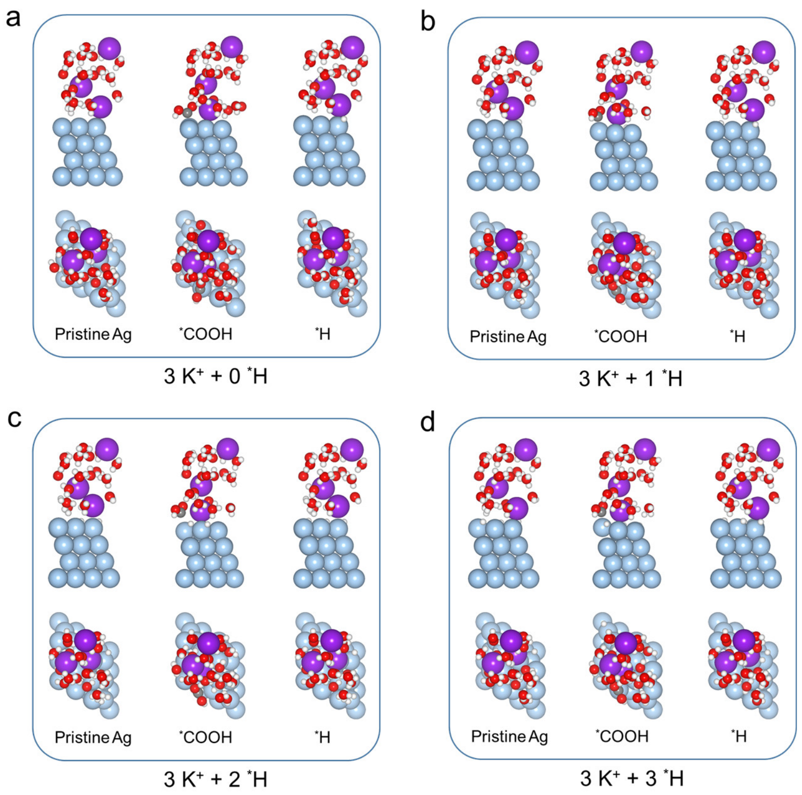

Supplementary Figure 35. The optimized periodic atomic structures under various *H coverages (1/9 per site), **a**, 3 K^+ + 0 *H, **b**, 3 K^+ + 1 *H, **c**, 3 K^+ + 2 *H and **d**, 3 K^+ + 3 *H. The light blue, purple, red, gray and white balls represent Ag, K, O, C and H, respectively.

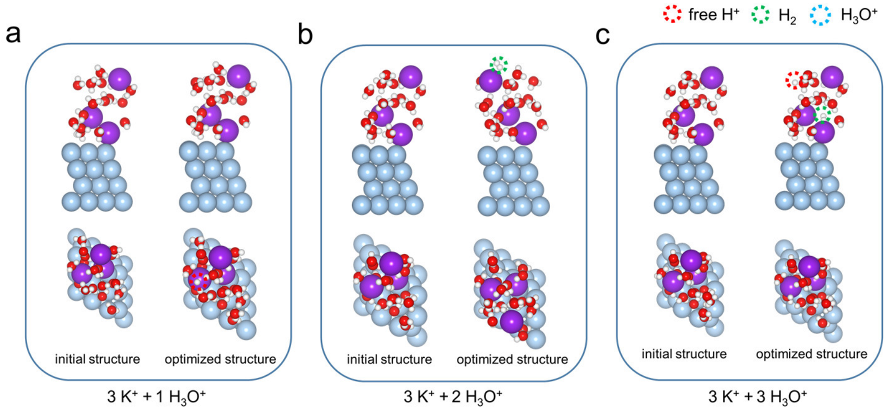

Supplementary Figure 36. The initial and optimized periodic atomic structures of 3 K^+ (1/18 per H_2O molecule) and different H_3O^+(1/18 per H_2O molecule). The light blue, purple, red, gray and white balls represent Ag, K, O, C and H, respectively.

We tried to use solvated H_3O^+ instead of *H to represent of H^+ in electrolyte. We first replaced one of the water molecules with a H_3O^+ in the model of 3 K^+ (1/18 per H_2O molecule). However, in the process of structure optimization, it was found that the H_3O^+ could not exist stably, and it would automatically evolve into a H_2O and a free H^+ (Supplementary Fig. 36). When we tried to replace two of water molecules with two H_3O^+, two free H^+ spontaneously form one molecule of H_2, and when we initially used three H_3O^+, the result was one molecule of H_2 and one free H^+. We reasoned that this is due to the presence of high concentration solvated K^+, the initial setting of H_3O^+ would be spontaneously separated from a free H^+ due to the steric hindrance and electrostatic repulsion effect[8,9]. When the free H^+ (from H_3O^+) in the electrolyte increased, two free H^+ spontaneously coupled to form one molecule of H_2.

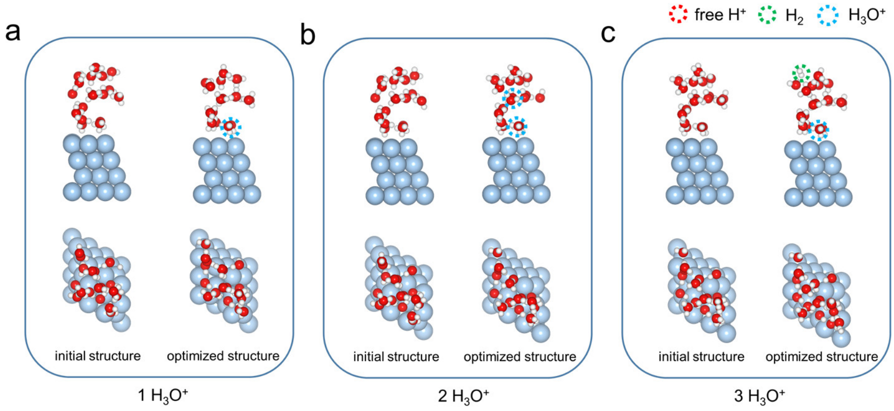

Supplementary Figure 37. The initial and optimized periodic atomic structures of different H_3O^+ (1/18 per H_2O molecule). The light blue, purple, red, gray and white balls represent Ag, K, O, C and H, respectively.

We removed all the K^+ of model (Supplementary Fig. 37), and found that no matter whether the H_3O^+ was one or two, it could exist stably. However, when the number of H_3O^+ increased to three, due to the steric hindrance effect of each other, a molecule of H_2 would spontaneously form and a H_3O^+ will be left. Therefore, it is high challenging to model the coexistence of high concentration solvated K^+ and H_3O^+ in electrolyte at the same time.

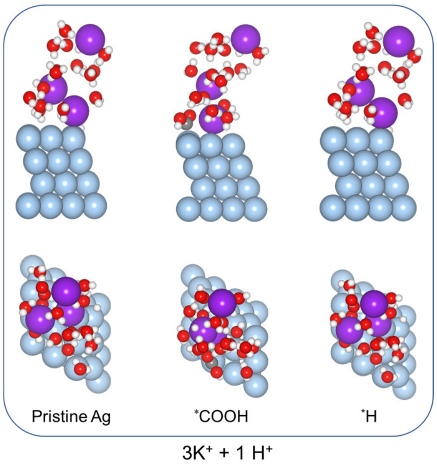

Supplementary Figure 38. The optimized periodic atomic structures of 3 K^+ (1/18 per H_2O molecule) and 1 H^+. The light blue, purple, red, gray and white balls represent Ag, K, O, C and H, respectively.

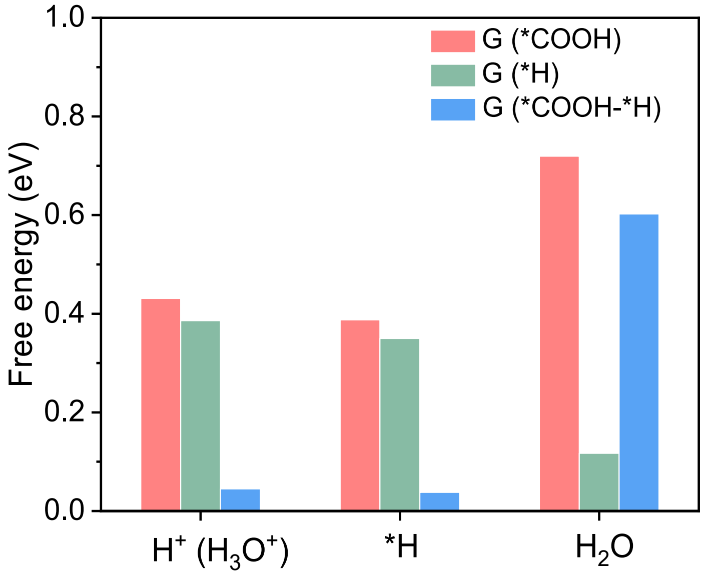

Supplementary Figure 39. The free energy for the formation of *COOH (G(*COOH)), *H (G(*H)), and their energy difference (G(*COOH)-G(*H)) on Ag (111) plane at 3 K^+ (1/18 per H_2O molecule) with different hydrogen sources.

Supplementary Table 1. Electrocatalytic performances for CO_2 to CO over typical recently reported catalysts.

Catalysts	Electrolyte	E (V vs. RHE)	*j* (A/cm^2)	FE (%)	Stability	Ref.
CD-Ag HPE	3.0 M KCl/0.05 M H_2SO_4	-1.41	4.5	95.0	2 A/cm^2 (200 h)	This work
CD-Ag HPE	3.0 M KCl/0.01 M $KHCO_3$	-0.98	2.5	90.0	/	This work
Ag-TEL	0.5 M $KHCO_3$	4.50*	3.37	88.0	1.75 A/cm^2 (210 h)	10
Ag-TEL	0.5 M $KHCO_3$	3.50*	1.75	92.1	/	10
activated-Ag HF	1.5 M $KHCO_3$	-0.83	1.26	92.7	-0.83 V (170 h)	3
Ni-N_5-C	0.5 M $KHCO_3$	-2.40	1.23	99.6	-2.20 V (100 h)	11
Hg-CoTPP/NG	1.0 M $KHCO_3$	-0.74	1.21	98.9	1.0 A/cm^2 (48 h)	12
Cl-Ag HF	3.0 M KCl	-0.91	1.0	92.2	1.0 A/cm^2 (150 h)	4
$Zn^{\delta+}$-NC	1.0 M KOH	/	1.00	99.0	0.5 A/cm^2 (1.5 h)	13
Ag/GDE	H_2O/10 mM CsOH	4.0 V*	0.867	76.0	0.5 A/cm^2 (100 h)	14
Ni-N-C	1.0 M KOH	-1.18	0.80	91.0	0.022 A/cm^2 (70 h)	15
Ag NPs	0.1 M CsOH/CsOH	3.4 V**	0.74	85.0	0.5 A/cm^2 (100 h)	16
Ag NPs	0.5 M KOH/CsOH	3.5 V**	0.72	90.0	0.45 A/cm^2 (200 h)	17
Ni-N_4/C-NH_2	1 M KOH	-1.00	0.526	85.0	-1.00 V (6 h)	18
Ag-NOLI	1.0 M $KHCO_3$	-0.86	0.50	84.0	0.2 A/cm^2 (6 h)	19
Ag-NOLI	1.0 M $KHCO_3$	-0.84	0.40	92.6	/	19

*The given potential is cell voltage.

** The given potential is cell voltage, $T_{Cathode}$ = 60 °C.

Supplementary Table 2. Detailed CO_2RR performances of CD-Ag HPE in CO_2-saturated 3 M KCl + 0.05 M H_2SO_4 (pH = 1, input CO_2 flow rate: 30 sccm).

j (A/cm^2)	E (V vs. RHE)	Mean FE_{CO} (%)	Standard deviation (%)	Mean FE_{H2} (%)	Standard deviation (%)	CO formation rate (mmol/h/cm^2)	CO energy efficiency (%)	Stability (h)
0.1	-0.42	0.88	± 0.11	99.21	± 0.20	0.02	0.71	/
0.2	-0.60	24.42	± 4.31	76.69	± 3.58	0.90	17.44	/
0.3	-0.68	87.40	± 1.46	13.73	± 2.85	4.89	60.48	/
0.4	-0.71	96.91	± 1.81	3.09	± 1.80	7.33	66.94	/
0.5	-0.75	98.92	± 0.74	1.33	± 0.54	9.35	66.95	/
0.8	-0.80	98.94	± 1.15	1.90	± 1.15	14.86	64.91	/
1.0	-0.84	98.15	± 1.30	1.84	± 1.32	18.55	63.54	/
1.2	-0.89	97.28	± 2.49	2.52	± 1.21	22.01	61.36	/
1.5	-0.93	97.79	± 1.57	2.93	± 1.20	27.60	60.42	/
1.8	-0.97	96.70	± 2.05	3.22	± 2.19	32.89	58.95	/
2.0	-1.01	96.78	± 0.97	3.68	± 1.30	36.57	57.89	200
2.5	-1.08	97.55	± 1.22	3.69	± 1.79	45.70	56.12	/
2.8	-1.13	96.47	± 2.32	3.58	± 2.27	51.04	54.89	/
3.0	-1.17	97.07	± 1.90	4.81	± 1.62	54.57	53.75	/
3.5	-1.24	95.34	± 2.03	4.75	± 2.06	63.05	51.72	/
3.8	-1.30	95.40	± 2.37	4.66	± 2.52	68.50	50.57	/
4.0	-1.34	95.98	± 2.05	3.59	± 1.39	71.94	49.63	/
4.5	-1.41	95.06	± 1.40	5.03	± 1.32	80.83	48.25	/
5.0	-1.49	92.60	± 3.53	7.87	± 4.32	86.73	45.23	/

Supplementary Table 3. The accuracy of the Alicat® mass flowmeter during CO_2 electroreduction tests performed at pH = 1

Entry	Conditions [a]	Corrected v_{outlet} [b] (sccm)	Accuracy of Alicat® mass flowmeter (sccm)			Deviation (%) [c]
			0.8% of reading	0.2% of full scale	total	
1	v_{CO2} = 30 sccm, j = 2 A/cm^2	30.27	± 0.24	± 0.10	± 0.34	± 1.12
2	v_{CO2} = 25 sccm, j = 2 A/cm^2	25.31	± 0.20	± 0.10	± 0.30	± 1.19
3	v_{CO2} = 20 sccm, j = 2 A/cm^2	20.37	± 0.16	± 0.10	± 0.26	± 1.28
4	v_{CO2} = 15 sccm, j = 2 A/cm^2	15.42	± 0.12	± 0.10	± 0.22	± 1.43
5	v_{CO2} = 10 sccm, j = 2 A/cm^2	10.81	± 0.09	± 0.10	± 0.19	± 1.76
6	v_{CO2} = 5 sccm, j = 2 A/cm^2	8.75	± 0.07	± 0.10	± 0.17	± 1.97

[a] Inlet flow rates were set and controlled by the Alicat® mass flow controller, and the constant j was applied by the Biologic VMP3 potentiostat.
[b] Readings of the outlet flow rate detected by the Alicat® mass flowmeter.
[c] Deviations were calculated from the total accuracy of mass flowmeter with the corrected v_{outlet}.

Supplementary Table 4. The calibrations of flow rate under representative test conditions at pH = 1.

Conditions		GC data		Outlet composition [c]	Uncorrected V_{outlet} [b]	Corrected V_{outlet} [b]	Deviation (%)
j (A/cm^2)	V_{CO2} [a] (sccm)	C_{CO} (ppm)	C_{H2} (ppm)	($CO/H_2/CO_2$, %)	(sccm)	(sccm)	
0	30	0	0	0/0/100	29.98	29.98	/
0.1	30	113	12488	0.01/1.2/98.8	30.38	30.51	0.43
2	10	655754	62490	65.6/6.2/28.2	10.67	10.81	1.30
2	5	522109	397093	50.2/42.0/7.8	8.62 [e]	8.75 [e]	1.49
2 [d]	5	1813	603254	0.2/60.3/39.5	12.61 [e]	12.78 [e]	1.33

tandard gas compositio [c] ($CO/H_2/CO_2$, %)	V_{input} [a] (sccm)	Measured V_{outlet} [b] (sccm)	Deviation (%)
0/0/100	30	29.96	0.13
0.1/1.0/98.9	30	30.07	0.23
65.2/5.8/29.0	10	9.97	0.30
50.2/40.8/9.0	5	5.02	0.40
0.5/60.2/39.3	5	4.99	0.20

[a] Inlet flow rates were set and controlled by Alicat® mass flow controller.
[b] Readings of the outlet flow rate detected by the Alicat® mass flowmeter.
[c] All standard gas mixtures flowed through the aqueous electrolyte solution at 25 °C.
[d] The CO_2 electroreduction test was performed at pH = 0.
[e] The actual measured outlet flow rate was higher than the inlet flow rate due to HER occurrence.

Supplementary Table 5. The Comparison between direct quantification by chromatography and calculation of unreacted CO_2 content of exhaust gas from electrolysis cell by formula method.

Conditions[a]	CO_2 concentration[b] (%)	CO_2 concentration[c] (%)	Deviation (%)
v_{CO2} = 30 sccm, j = 2 A/cm^2	74.8	74.4	0.5
v_{CO2} = 10 sccm, j = 2 A/cm^2	28.2	27.8	1.4
v_{CO2} = 5 sccm, j = 2 A/cm^2	7.8	8.0	2.5

[a] The CO_2 electroreduction test was performed at pH = 1.
[b] The CO_2 concentration was calculated by formula based on GC quantification of CO and H_2.
[c] The CO_2 concentration was quantified by online GC.

Supplementary Table 6. Comparison of the stable *j* and CO_2 SPCE of CD-Ag HPE with those of benchmark alkaline, neutral and acidic CO_2RR.

Catalysts	Electrolyte	pH	*j* (A/cm^2)	CO_2 SPCE (%)	Stability	Ref.
CD-Ag HPE	3.0 M KCl/0.05 M H_2SO_4	1	2.0	86.6	2 A/cm^2 (200 h)	This work
Ag GDE	0.01 M H_2SO_4/0.01 M Cs_2SO_4	1.7	0.06	90	0.06 A/cm^2 (50 h)	37
Ni-N-C	K_2SO_4/H_2SO_4	0.5	0.6	85	0.5 A/cm^2 (10 h)	31
Cu PCRL	0.01 M H_2SO_4	1.7	0.1	85	0.1 A/cm^2 (9 h)	34
Cu/PFSA	3.0 M KCl/1 M H_3PO_4	< 1	1.2	77	1.2 A/cm^2 (12 h)	6
PTFE-Cu	3.0 M KCl/H_3PO_4	1	0.2	75	0.2 A/cm^2 (30 h)	35
Bi_2S_3-derived	1.0 M KOH	> 14	2.0	67	0.25 A/cm^2 (100 h)	25
Pd–Cu/PTFE	0.5 M K_2SO_4/H_2SO_4	2	0.5	60	0.6 A/cm^2 (4.5 h)	38
ER-CuNS	3.0 M KCl/0.05 M H_2SO_4	1	0.56	54.4	~0.7 A/cm^2 (30 h)	21
activated-Ag HF	1.5 M $KHCO_3$	8.2	1.26	54.1	-0.83 V (170 h)	3
Hg-CoTPP/NG	1.0 M $KHCO_3$	8.0	1.21	45.6	1.0 A/cm^2 (48 h)	12
Bi HF	2.0 M $KHCO_3$	8.3	1.042	36.6	-1.01 V (40 h)	29
Bi/C/PTFE	1.0 M KOH	> 14	0.815	34.6	-0.6 V (6 h)	36
nBuLi-Bi	1.0 M $KHCO_3$	8.0	0.46	29.1	0.03 A/cm^2 (100 h)	32
BiNS	3.0 M KCl/0.05 M H_2SO_4	1	0.257	27.4	-1.23 V (8 h)	7
s-SnLi	2.0 M KOH	> 14	1.012	23.5	0.12 A/cm^2 (150 h)	30
F-Cu	0.5 M KOH	> 14	1.6	19.3	0.4 A/cm^2 (40 h)	28
Ni-N_5-C	0.5 M $KHCO_3$	7.2	1.23	18.7	-2.20 V (100 h)	11
$Sn_{2.7}Cu$	1.0 M KOH	> 14	0.40	18.51	-0.55 V (40 h)	33

Supplementary Table 7. pH values of different CO_2-saturated catholytes.

Electrolyte	pH
0.5 M H_2SO_4 + 3.0 M KCl	0
0.15 M H_2SO_4 + 3.0 M KCl	0.5
0.05 M H_2SO_4 + 3.0 M KCl	1
0.005 M H_2SO_4 + 3.0 M KCl	2
0.0005 M H_2SO_4 + 3.0 M KCl	3
3.0 M KCl	4
3.0 M KCl + 0.01 M $KHCO_3$	6.6

Supplementary Table 8. EIS fitting parameters of different CO_2-saturated catholytes by using same equivalent circuit.

Electrolyte	R_s (Ohm cm^2)	R_{ct} (Ohm cm^2)	C_{dl} (μF cm^{-2})
0.05 M H_2SO_4 + 0 M KCl	8.9	2.7	587
0.05 M H_2SO_4 + 0.1 M KCl	5.9	1.8	631
0.05 M H_2SO_4 + 0.5 M KCl	2.0	1.3	711
0.05 M H_2SO_4 + 1.0 M KCl	1.1	1.1	726
0.05 M H_2SO_4 + 3.0 M KCl	0.5	0.9	905

Supplementary Table 9. The solution resistances of CO_2-saturated 3 M KCl + 0.05 M H_2SO_4 catholytes at different j.

j (A/cm^2)	Solution resistance (Ohm cm^2)
OCV	0.52
0.1	0.54
0.5	0.50
1.0	0.47
1.5	0.51
2.0	0.48
2.5	0.53
3.0	0.49
4.0	0.47
5.0	0.52

Supplementary Table 10. The amount of dissolved Ag in the outlet electrolyte quantified by ICP-OES.

Reaction time	Dissolved Ag
0.5 h (OCP)	Not detected
1.0 h (stability test)	Not detected
20 h (stability test)	Not detected
50 h (stability test)	Not detected
100 h (stability test)	Not detected
200 h (stability test)	Not detected

Supplementary Table 11. Diffusion coefficient for different species.

Species	*D* [10^{-9} m^2/s]
H^+	9.311
K^+	1.957
OH^-	5.293
HCO_3^-	1.185
CO_3^{2-}	0.923
SO_4^{2-}	1.065
Cl^-	1.185
CO_2	1.910

Supplementary References

1 Gu, J. *et al.* Modulating electric field distribution by alkali cations for CO_2 electroreduction in strongly acidic medium. *Nat. Catal.* **5**, 268-276 (2022).

2 Qin, H.-G. *et al.* Quantitative understanding of cation effects on the electrochemical reduction of CO_2 and H^+ in acidic solution. *ACS Catal.* **13**, 916-926 (2023).

3 Li, S. J. *et al.* Hierarchical micro/nanostructured silver hollow fiber boosts electroreduction of carbon dioxide. *Nat. Commun.* **13**, 3080 (2022).

4 Li, S. J. *et al.* Chloride ion adsorption enables ampere-level CO_2 electroreduction over silver hollow fiber. *Angew. Chem. Int. Ed.* **61**, e202210432 (2022).

5 Hsieh, Y. C., Senanayake, S. D., Zhang, Y., Xu, W. Q. & Polyansky, D. E. Effect of chloride anions on the synthesis and enhanced catalytic activity of silver nanocoral electrodes for CO_2 electroreduction. *ACS Catal.* **5**, 5349-5356 (2015).

6 Huang, J. E. *et al.* CO_2 electrolysis to multicarbon products in strong acid. *Science* **372**, 1074-1078 (2021).

7 Qiao, Y. *et al.* Engineering the local microenvironment over Bi nanosheets for highly selective electrocatalytic conversion of CO_2 to HCOOH in strong acid. *ACS Catal.* **12**, 2357-2364 (2022).

8 Chen, L. D., Urushihara, M., Chan, K. R. & Norskov, J. K. Electric field effects in electrochemical CO_2 reduction. *ACS Catal.* **6**, 7133-7139 (2016).

9 Resasco, J. *et al.* Promoter effects of alkali metal cations on the electrochemical reduction of carbon dioxide. *J. Am. Chem. Soc.* **139**, 11277-11287 (2017).

10 Wen, G. *et al.* Continuous CO_2 electrolysis using a CO_2 exsolution-induced flow cell. *Nat. Energy* **7**, 978-988 (2022).

11 Huang, J.-R. *et al.* Single-product faradaic efficiency for electrocatalytic of CO_2 to CO at current density larger than 1.2 A cm^{-2} in neutral aqueous solution by a single-atom nanozyme. *Angew. Chem. Int. Ed.* **61**, e202210985 (2022).

12 Fang, M., Xu, L., Zhang, H., Zhu, Y. & Wong, W.-Y. Metalloporphyrin-linked mercurated graphynes for ultrastable CO_2 electroreduction to CO with nearly 100% selectivity at a current density of 1.2 A cm^{-2}. *J. Am. Chem. Soc.* **144**, 15143-15154 (2022).

13 Li, S. *et al.* Low-valence $Zn^{\delta+}$ ($0<\delta<2$) single-atom material as highly efficient electrocatalyst for CO_2 reduction. *Angew. Chem. Int. Ed.* **60**, 22826-22832 (2021).

14 Ye, K. *et al.* Resolving local reaction environment toward an optimized CO_2-to-CO conversion performance. *Energy Environ. Sci.* **15**, 749-759 (2022).

15 Li, Y. *et al.* Atomically dispersed single Ni site catalysts for high-efficiency CO_2 electroreduction at industrial-level current densities. *Energy Environ. Sci.* **15**, 2108-2119 (2022).

16 Endrodi, B. *et al.* High carbonate ion conductance of a robust PiperION membrane allows industrial current density and conversion in a zero-gap carbon dioxide electrolyzer cell. *Energy Environ. Sci.* **13**, 4098-4105 (2020).

17 Endrődi, B. *et al.* Operando cathode activation with alkali metal cations for high current density operation of water-fed zero-gap carbon dioxide electrolysers. *Nat. Energy* **6**, 439-448 (2021).

18 Chen, Z. *et al.* Amination strategy to boost the CO_2 electroreduction current density of M–N/C single-atom catalysts to the industrial application level. *Energy Environ. Sci.* **14**, 2349-2356 (2021).

19 Kim, D. *et al.* Selective CO_2 electrocatalysis at the pseudocapacitive nanoparticle/ordered-ligand interlayer. *Nat. Energy* **5**, 1032-1042 (2020).

20 De Arquer, F. P. G. *et al.* CO_2 electrolysis to multicarbon products at activities greater than 1 A cm^{-2}. *Science* **367**, 661-666 (2020).

21 Ma, Z. *et al.* CO_2 electroreduction to multicarbon products in strongly acidic electrolyte via synergistically modulating the local microenvironment. *Nat. Commun.* **13**, 7596 (2022).

22 Zheng, M. *et al.* Electrocatalytic CO_2-to-C_{2+} with ampere-level current on heteroatom-engineered copper via tuning *CO intermediate coverage. *J. Am. Chem. Soc.* **144**, 14936-14944 (2022).

23 Zhao, Y. *et al.* Industrial-current-density CO_2-to-C_{2+} electroreduction by anti-swelling anion-exchange ionomer-modified oxide-derived Cu nanosheets. *J. Am. Chem. Soc.* **144**, 10446-10454 (2022).

24 Shen, H. *et al.* In-situ constructuring of copper-doped bismuth catalyst for highly efficient CO_2 electrolysis to formate in ampere-level. *Adv. Energy Mater.* **13**, 2202818 (2022).

25 Lin, L. *et al.* A nanocomposite of Bi clusters and $Bi_2O_2CO_3$ sheets for highly efficient electrocatalytic reduction of CO_2 to formate. *Angew. Chem. Int. Ed.* **62,** e202214959 (2022).

26 Zhang, L. *et al.* Atomically dispersed Ni-Cu catalysts for pH-universal CO_2 electroreduction. *Adv. Mater.* **35**, 2209590 (2023).

27 Li, L., Liu, Z., Yu, X. & Zhong, M. Achieving high single-pass carbon conversion efficiencies in durable CO_2 electroreduction in strong acids via electrode structure engineering. *Angew. Chem. Int. Ed.* **62**, e202300226 (2023).

28 Ma, W. C. *et al.* Electrocatalytic reduction of CO_2 to ethylene and ethanol through hydrogen-assisted C-C coupling over fluorine-modified copper. *Nat. Catal.* **3**, 478-487 (2020).

29 Chen, A. *et al.* Gas penetrating hollow fiber Bi with contractive bond enables industry-level CO_2 electroreduction. *Appl. Catal. B-Environ.* **333**, 122768 (2023).

30 Yan, S. *et al.* Electron localization and lattice strain induced by surface lithium doping enable ampere-level electrosynthesis of formate from CO_2. *Angew. Chem. Int. Ed.* **60**, 25741-25745 (2021).

31 Li, H. *et al.* Tailoring acidic microenvironments for carbon-efficient CO_2 electrolysis over Ni-N-C catalyst in a membrane electrode assembly electrolyzer. *Energy Environ. Sci.* **16**, 1502-1510 (2023).

32 Fan, L., Xia, C., Zhu, P., Lu, Y. Y. & Wang, H. T. Electrochemical CO_2 reduction to high-concentration pure formic acid solutions in an all-solid-state reactor. *Nat. Commun.* **11**, 3633 (2020).

33 Ye, K. *et al.* In situ reconstruction of a hierarchical Sn-Cu/SnO_x core/shell catalyst for high-performance CO_2 electroreduction. *Angew. Chem. Int. Ed.* **59**, 4814-4821 (2020).

34 O'Brien, C. P. *et al.* Single pass CO_2 conversion exceeding 85% in the electrosynthesis of multicarbon products via local CO_2 regeneration. *ACS Energy Lett.* **6**, 2952-2959 (2021).

35 Zhao, Y. *et al.* Conversion of CO_2 to multicarbon products in strong acid by controlling the catalyst microenvironment. *Nat. Synth.* **2**, 403-412 (2023).

36 Xing, Z., Hu, X. & Feng, X. F. Tuning the microenvironment in gas-diffusion electrodes enables high-rate CO_2 electrolysis to formate. *ACS Energy Lett.* **6**, 1694-1702 (2021).

37 Pan, B. *et al.* Close to 90% single-pass conversion efficiency for CO_2 electroreduction in an acid-fed membrane electrode assembly. *ACS Energy Lett.* **7**, 4224-4231 (2022).

38 Xie, Y. *et al.* High carbon utilization in CO_2 reduction to multi-carbon products in acidic media. *Nat. Catal.* **5**, 564-570 (2022).
